# All In: A Casino-Themed Emergency Medicine Conference Day

**DOI:** 10.5070/M5.52394

**Published:** 2026-10-31

**Authors:** Shorok Hassan, Danielle Langan, Amar Bukvic, Moshe Weizberg, Barry Hahn

**Affiliations:** * Northwell, New Hyde Park, NY; ^ Department of Emergency Medicine, Staten Island University Hospital, Staten Island, NY; † Department of Emergency Medicine, Zucker School of Medicine at Hofstra/Northwell, Hempstead, NY; ** Department of Emergency Medicine, Maimonides Health, NY

## Abstract

**Audience:**

The target audience includes individuals involved in the education and training of emergency medicine (EM) residents, such as program directors, assistant program directors, core faculty, and academic attendings, as well as learners including residents, physician assistant (PA) fellows, and medical students (MS).

**Introduction:**

Gamification has increasingly been incorporated into emergency medicine (EM) residency education to enhance learner engagement, motivation, and active participation. Prior studies have demonstrated benefits of game-based learning across multiple areas of medical education, including improved knowledge retention, teamwork, communication, and learner satisfaction.^1^–^4^ However, most gamification interventions in EM involve isolated activities rather than comprehensive, multi-station conference curricula. To address this gap, we developed a casino-themed, multi-station educational conference designed to reinforce core EM knowledge, procedural skills, and clinical reasoning through competitive, team-based learning. The intervention incorporated simulation, rapid-response scenarios, board-style trivia, and wagering poker chips during a protected half-day residency conference.

**Educational Objectives:**

By the end of this educational intervention, participants will be able to: 1) demonstrate airway management skills through simulation, including equipment setup and timely interventions for both adult and pediatric cases, 2) interpret various electrocardiograms (EKGs) in high-pressure scenarios, 3) analyze cases and apply evidence-based management in various rapid-response scenarios, 4) demonstrate rapid recall of EM knowledge by answering board-style trivia questions, and 5) demonstrate increased confidence in team-based decision-making, effective communication under pressure, and leadership in high-stress emergency situations.

**Educational Methods:**

This prospective educational innovation was delivered as a single, four-hour casino-themed EM conference session featuring interactive stations modeled after casino games and held during the residency program’s weekly Wednesday educational conference. The session featured interactive, competition-based stations designed to replicate high-pressure ED scenarios. Learners rotated through four game-based stations: “Play Your Cards Right” for rapid-recall trivia using EM board-style questions, “Diagnostic Craps” for EKG interpretation and management, “High Stakes Airways” for hands-on airway management using mannequins and rapid sequence intubation scenarios, and “Rapid Response Roulette” for discussion of ED cases focusing on team dynamics, leadership, resuscitation, and escalation of care. The games were designed to reflect real-world ED challenges, with content and expectations tailored to postgraduate years (PGY) levels. Participants wagered poker chips based on confidence in their responses, reinforcing self-assessment and accountability. This method encouraged active participation, peer-to-peer learning, and application of clinical reasoning in high-pressure scenarios.

**Research Methods:**

The casino-style conference featured six tables each with an EM attending facilitator. Participants included EM residents, physician assistant (PA) fellows, and medical students. Learners started with 15 poker chips and engaged in four game-based sessions over four hours. The games “Play Your Cards Right,” “High Stakes Airways,” “Diagnostic Craps,” and “Rapid Response Roulette” incorporated either case-based simulation, procedural skill stations, rapid decision-making, EKG interpretation, and/or evidence-based medicine (EBM) trivia. Learners wagered chips based on confidence, with gains/losses tied to performance. Faculty provided real-time feedback and facilitated discussions. Pre- and post-conference surveys (Appendix A) assessed educational value, skill improvement, confidence, and clinical applicability. Instructors also evaluated feasibility and effectiveness. The study was Institutional Review Board exempt.

**Results:**

Of the 33 learners who attended the conference, 32 completed surveys (97% response rate) including 26 EM residents (10 PGY-1, 8 PGY-2 and 8 PGY-3), 4 medical students, and 2 physician assistants. One hundred percent of participants answered “yes” when asked if they would recommend this type of conference to peers and 100% preferred this learning style to traditional didactics. Eighty-eight percent of the respondents reported they are “very likely” to incorporate skills learned from this activity into future practice.

Facilitators rated the game-based learning activities as highly effective teaching tools, with 92% rating 4 or 5 (on a 5-point scale). Facilitators were also asked if they found the games easy to implement, and 83% answered rating a 4 or a 5 (on a 5-point scale). Responses derived from the open-ended questions emphasize the conference’s effectiveness in engaging participants, providing meaningful learning experiences and fostering a positive and well-organized environment.

**Discussion:**

This casino-style, game-based learning conference was highly rated for both educational value and engagement. Participants reported increased confidence in managing emergency scenarios, particularly in EKG interpretation and team-based critical decision-making. The format was viewed as feasible and effective, with most participants preferring game-based learning over traditional didactics, supporting its continued use in EM education.

**Topics:**

Gamification, EM education, simulation, active learning, residency training.

## USER GUIDE

**Table:** 

List of Resources:	
Abstract	81
User Guide	84
Small Groups Learning Materials	93
Appendix A: Surveys	96
Appendix B: Standardized Facilitator Guide	104
sGAE Questions	110
sGAE Answers	124

**Learner Audience:**

Individuals involved in the education and training of emergency medicine (EM) residents, such as program directors, assistant program directors, core faculty, and academic attendings, as well as learners including residents, physician assistant (PA) fellows, and medical students (MS).

**Time Required for Implementation:**

5 hours:

1 hour for preparation and instructions

1 hour per game (4 hours for 4 games)

**Recommended Number of Learners Per Instructor:**

~6 learners per instructor

**Topics:**

Gamification, EM education, simulation, active learning, residency training.

**Objectives:**

By the end of this educational intervention, participants will be able to:

1. Demonstrate procedural competence and clinical decision-making in airway management by engaging in simulation-based scenarios that require accurate equipment setup and timely execution of appropriate airway interventions for both pediatric and adult emergency cases.
2. Interpret various EKGs in high-pressure scenarios.
3. Analyze clinical information and apply evidence-based management strategies by systematically working through emergency department (ED) cases and answering targeted questions in high-acuity, rapid-response scenarios.
4. Demonstrate rapid recall of EM knowledge by answering board-style trivia questions, reinforcing critical concepts through competitive gameplay.
5. Demonstrate increased confidence in team-based decision-making, effective communication under pressure, and leadership in high-stress emergency situations.

### Linked objectives and methods

As graduate medical education (GME) evolves, EM residencies have embraced gamification, the use of game mechanics in non-game contexts, as a strategy to enhance learner engagement, motivation, and problem-solving skills.^1^ Gamification leverages competition, rewards, and interactive elements to transform passive learning into dynamic, active experiences, aligning with the fast-paced, high-stakes nature of EM.

Prior studies have demonstrated the benefits of gamified approaches across various domains of medical education. For example, gamification has been successfully applied to disaster medicine, obstetrics and gynecology, and point-of-care ultrasound (POCUS) education, improving knowledge retention, learner enjoyment, communication, and teamwork.^2^ The Cochrane review by Akl et al. highlighted that educational games can positively influence health professionals’ knowledge and skills compared to traditional teaching.^3^ Additionally, the Society for Academic Emergency Medicine (SAEM) and the Academy of Emergency Ultrasound (AEUS) have formally supported integrating gamification into EM training, recognizing its potential to enhance educational outcomes.^4^ Despite these advances, most existing implementations focus on single-session or isolated game interventions rather than comprehensive, multi-station curricula embedded within standard residency conferences.

This study addresses a gap in EM education by implementing a multi-station, casino-themed gamification conference designed to reinforce core EM concepts, procedural skills, and clinical reasoning through competitive, team-based learning. The intervention, which incorporated wagering poker chips, was delivered during a protected half-day residency conference. We evaluated feasibility, participant reception, and perceived educational value, with the goal that this approach would enhance learner engagement, increase self-reported confidence in EM skills, and encourage application of these skills in clinical practice.

Each learning objective was embedded within a game designed to reflect realistic ED challenges. For example, Objective 1 focused on procedural competency in airway management, which was achieved using hands-on airway simulation stations utilizing mannequins, procedural checklists, and time-sensitive airway scenarios requiring learners to select medications, prepare equipment, and perform airway interventions at the “High Stakes Airways” station. A pre-established checklist (Council of Residency Directors [CORD] Standardized Direct Observational Assessment Tools [SDOTS]) for endotracheal intubation was used by the facilitators during this activity.

Clinical reasoning and diagnostic accuracy were reinforced at “Diagnostic Craps,” requiring learners to identify and interpret emergent cardiac rhythms and determine appropriate next-step management strategies (Objective 2). For these games, content was tailored to match baseline competencies with questions created for specific PGY levels.

In “Rapid Response Roulette,” the learners had to analyze clinical information randomly presented to them in the form of high-acuity rapid-response scenarios and correctly identify critical “next steps,” emphasizing real-time decision-making and resuscitation leadership skills (Objective 3).

“Play Your Cards Right” showcased the learners’ ability to demonstrate recall of foundational and relevant EM knowledge (Objective 4) through casino-style trivia questions using board-review style focused on high-yield clinical topics. Participants were dealt cards using a deck of standard playing cards. Based on the card they received, they were asked specific board-style trivia questions.

Objective 5 was integrated throughout all four games through collaborative, team-based gameplay requiring communication, delegation, and clinical decision-making.

### Recommended pre-reading for facilitator

Standardized facilitator guides: Includes instructions and layout for the day as provided in Appendix B. The content for each game was provided in advance (see sGAE questions and answers). This includes EKG tracings and interpretation guides for “Play Your Cards Right,” clinical scenarios and management plans for “Rapid Response Roulette,” airway cases and procedural checklist for “High Stakes Airways,” and board-style trivia questions with answer keys for “Diagnostic Craps.”

### Small group application exercise (sGAE)

See the following attached materials for this small group exercise

- Appendix A: Surveys
- Appendix B: Standardized Facilitator Guide
- sGAE Questions
- sGAE Answers

### Results and tips for successful implementation

This educational innovation took place at Staten Island University Hospital (SIUH), a tertiary-care teaching hospital in New York City. The emergency department (ED) at SIUH supports an ACGME-accredited, three-year EM residency program with 10 residents per postgraduate year, totaling 30 EM residents across all training levels during the study period. The department is also staffed by approximately 25 certified physician assistants and operates a two-year PA post-graduate training program, enrolling two to three fellows annually. Both PA fellows and certified PAs regularly participate in the department’s weekly Wednesday EM conference, which provides protected time for the educational intervention.

There were six “casino” tables set up, with one facilitator per table. Based on anticipated attendance, learners were pre-assigned into six groups, one for each table. There were 6 learners per group except for the PGY-III groups, which had 4 learners per table. Groups were primarily organized by PGY level, while also incorporating the other learners (PA fellows and medical students) who regularly attend conferences based on their training level and comfort. Two tables included PGY-1 residents and PA fellows, two tables were assigned to PGY-2 residents, and two to PGY-3 residents. Medical students were intentionally dispersed among the PGY-2 tables to promote engagement and collaborative learning.

Six EM attending physicians, all experienced in medical education and simulation, facilitated the stations and completed post-event evaluations. Each table hosted four interactive games over the course of four hours.

### Participant Demographics

Thirty-three learners attended the casino-themed EM conference, which was conducted during the program’s weekly Wednesday educational conference. Of these, thirty-two (97%) completed both pre- and post-event surveys. The cohort consisted of 26 (81%) EM residents. Among these residents, ten were postgraduate year one (PGY-1), eight were postgraduate year two (PGY-2), and eight were postgraduate year three (PGY-3). Four medical students (13%) and two physician assistant fellows (6%) also attended the conference.

Additionally, six attending physicians completed postconference surveys evaluating the educational session. All faculty had expertise in bedside teaching, simulation, and small-group instruction, and they actively participated in planning and delivering the casino-themed conference.

### Pre-Conference Confidence and Learning Goals

Prior to the conference, learners reported varying levels of confidence in core EM skills. Fifteen learners (47%) rated their airway management experience as advanced, while one learner (3%) reported expert-level experience. Ten learners (31%) described themselves as having intermediate airway skills, and six (19%) felt they were at a novice level. Regarding confidence in making rapid diagnostic decisions, sixteen learners (50%) felt somewhat confident, eleven (34%) felt confident, four (13%) were not confident, and one (3%) was very confident. For exposure to rapid response scenarios, seventeen learners (53%) reported frequent encounters, ten (31%) reported occasional encounters, three (9%) reported rare encounters, and two (6%) reported very frequent encounters. For confidence interpreting electrocardiograms in emergencies, nineteen learners (59%) felt somewhat confident, seven (22%) felt confident, five (16%) felt not confident, and one (3%) felt very confident.

Learners identified areas they hoped to improve through the conference: airway management (47%), diagnostic skills (59%), team-based critical decision-making (56%), communication under pressure (75%), rapid response and emergency intervention (66%), electrocardiogram interpretation (88%), and leadership in high-stress situations (69%). Additionally, 38% of learners described themselves as moderately familiar with game-based or simulation-based learning prior to the conference (Table 1). Figure 1 illustrates the distribution of pre-conference learning goals among all participants.

### Learner-Reported Improvements

After the conference, learners rated educational activities highly overall. “Diagnostic Craps” was rated a 5 (extremely valuable) by 88% of learners, “Play Your Cards Right” by 84%, “Rapid Response Roulette” by 81%, and “High Stakes Airways” by 69%. Learners reported perceived improvements in key skills, with 91% indicating improved electrocardiogram interpretation, 81% improved team-based decision-making, 69% improved rapid response and emergency intervention, 66% improved airway management, 56% improved diagnostic skills, 50% improved communication under pressure, and 31% improved leadership in high-stress situations.

Regarding skills learners hoped to improve, fewer than half who aimed to improve leadership reported gains (32%). In contrast, most who hoped to improve EKG interpretation (93%), team-based decision-making (83%), and rapid response skills (76%) did report improvement. Nearly 78% of learners stated they were very likely to apply skills gained from the conference in their future clinical practice. Additionally, 59% felt significantly more confident managing high-stakes situations afterward, while 13% felt more confident overall. All participants (100%) reported they would recommend this conference format to peers, and all preferred it over traditional didactic teaching.

#### Faculty Observations

All six attending physicians completed post-conference surveys. Faculty unanimously identified electrocardiogram interpretation as the most effectively taught skill. All six respondents rated the teaching tools highly, with 92% assigning scores of 4 or 5 on a 5-point scale for overall effectiveness. Ease of implementation was rated 4 or 5 by 83% of faculty. “Play Your Cards Right” and “Rapid Response Roulette” received perfect scores for ease of facilitation from all respondents. Faculty also reported that diagnostic, decision-making, communication, and rapid response skills were effectively taught and learned. All faculty members agreed that game-based learning was more effective than traditional didactics for teaching specific EM skills and highlighted the interactive and competitive elements as the most successful aspects of the conference (Table 2).

### Open-Ended Feedback

Learner comments emphasized engagement, enjoyment, and perceived meaningful learning experiences, with multiple responses noting the well-organized environment. This qualitative feedback highlights the importance of effective conference logistics, clear instructions, timely rotations, and enthusiastic facilitation in maximizing educational benefits.

### Implementation and Improvement

This novel, casino-themed EM conference resulted in strong learner engagement and substantial perceived improvements in key emergency medicine skills, particularly EKG interpretation, team-based decision-making, and rapid response management. All participants preferred the format over traditional didactics, and nearly 90% reported they were very likely to apply the skills learned in future clinical practice. Faculty observations supported learner perceptions, identifying EKG interpretation and decision-making as the most effectively taught skills. The high attendance and response rate (97%) also demonstrated the feasibility of incorporating a multi-station, game-based format into routine residency conferences.

The intervention was well received across multiple training levels, including residents, medical students, and PA fellows, suggesting broad educational appeal. While learners reported notable gains in most targeted skills, improvements in leadership under stress were less pronounced, indicating that gamification alone may be less effective for teaching leadership competencies. These findings are consistent with longstanding recognition of the challenge of effectively teaching this complex skill during training.^6^

Compared to previous reports on gamification and simulation, this study uniquely combined multiple game-based learning activities into a cohesive, conference-wide intervention, using competitive elements such as wagering poker chips to foster engagement.^7^–^9^ Prior studies have demonstrated that singlegame interventions can improve short-term learner satisfaction and engagement. However, few have evaluated multi-game-based curricula integrated into scheduled residency conferences. The overwhelmingly positive learner and faculty responses in this study align with existing literature showing gamification enhances motivation and perceived learning but extend these findings by demonstrating high feasibility for implementation within a half-day academic conference.^10^

Key limitations included reliance on self-reported outcomes, lack of objective assessment of knowledge or skill acquisition, immediate post-session surveys that may reflect short-term enthusiasm, and a small single-institution sample limiting generalizability. Long-term retention and impact on clinical practice were also not assessed. Overall, the findings suggest that gamified, interactive educational strategies may enhance learner engagement, confidence, and perceived preparedness for high-acuity clinical scenarios in EM training.

### Implementation Tips

Educators considering a similar intervention should:

- Provide clear facilitator guides and standardized materials to promote consistency across multiple game stations.
- Incorporate debriefing focused not only on clinical content but also on communication, leadership dynamics, and teamwork strategies.
- Consider surveying learners in advance about their familiarity with competitive learning to tailor the format.

### Pearls

- **High Stakes Airways**
  ○ Tailor airway approach to the clinical scenario (i.e., C-spine use inline stabilization, aspiration in GI bleeds and drowning)^11^
  ○ Choose Rapid Sequence Intubation (RSI) drugs based on hemodynamics and disease process
  ○ Always have adjuncts and backups ready including bougie, suction, fiberoptics, cricothyroidotomy kit, etc.
  ○ Consider key ventilator strategies in different pathologies (i.e., low tidal volumes and low respiratory rates in asthmatics)^12^
- **Diagnostic Craps**
  ○ Recognize and interpret common EKG presentations that we see in the ED (i.e., STEMI, atrial fibrillation, atrial flutter, heart block, supraventricular tachycardia, hyperkalemia, ventricular tachycardia, Brugada syndrome, Wolf-Parkinson-White, and de-Winter T waves)
- **Play Your Cards Right**
  ○ Recognize life-threatening conditions and recall their first-line interventions in adult and pediatric EM patients (i.e., status epilepticus, thyroid storm, electrolyte derangements, intussusception, epiglottitis)
  ○ Understand high yield toxicology treatments and antidotes (i.e., naloxone, *N*-Acetylcysteine (NAC), fomepizole, deferoxamine, physostigmine)
  ○ Apply clinical guidelines in practice when applicable (i.e., Kocher for septic arthritis, Rumack-Matthew nomogram for acetaminophen overdose)
- **Rapid Response Roulette**
  ○ Recognize abnormal vital signs and high-acuity cases and identify immediate next step actions (i.e., intubation in burn patient with airway compromise, epinephrine for anaphylaxis, antibiotics and surgery consultation for necrotizing fasciitis)
  ○ Identify decompensating patients (hypotension, hypoxia, altered mental status)
  ○ Prioritize first steps including ABCs, IV access, oxygen, glucose
  ○ Be able to describe and perform procedures (i.e., lumbar puncture, pericardiocentesis, shoulder reduction, T-piece ventilation, lateral canthotomy, pigtail catheter, needle cricothyrotomy)
  ○ Have a wide differential diagnosis but tailor next step actions to the most likely cause

**Figure:**
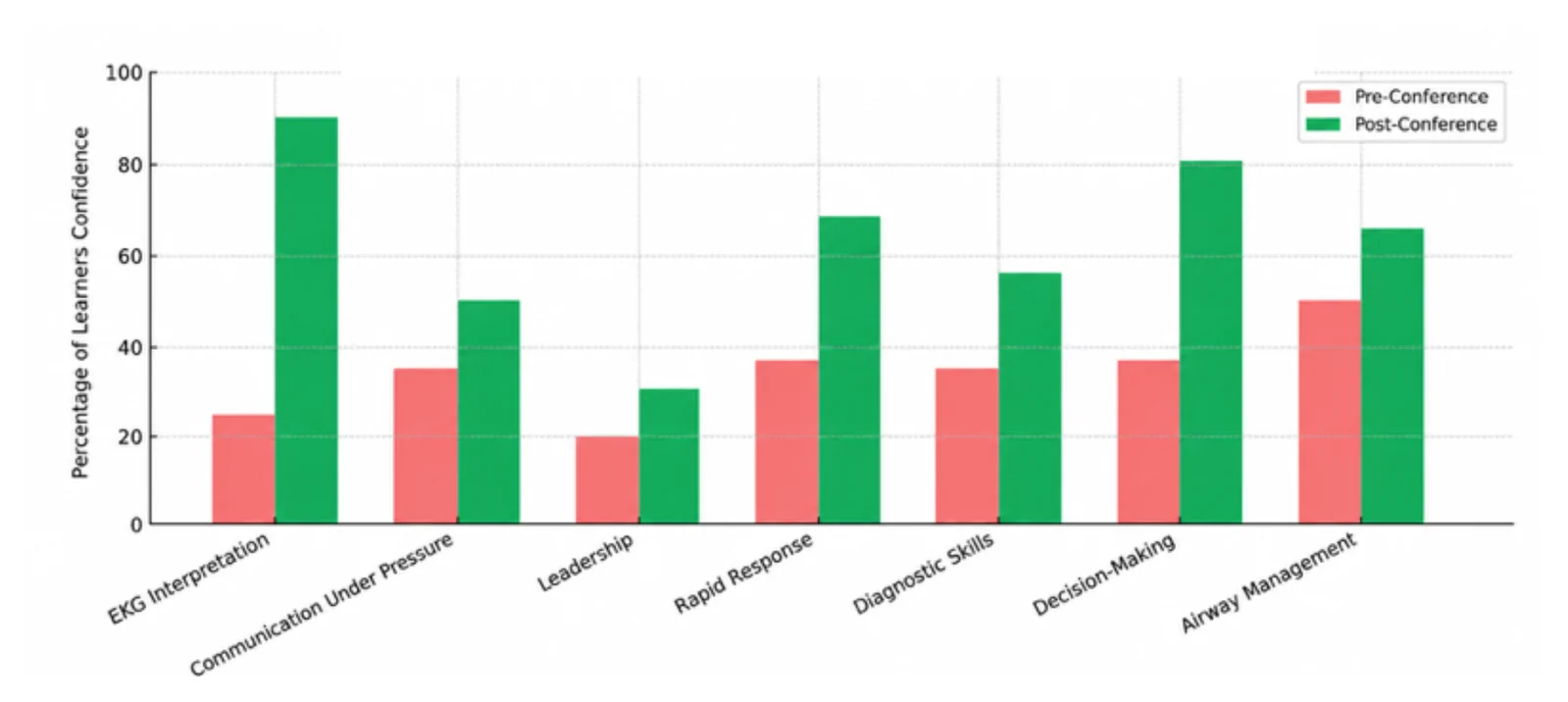
Learner Confidence in Learning Goals: Pre- vs. Post-Conference

**Figure:**
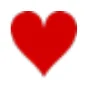


**Figure:**
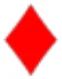


**Figure:**
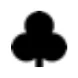


**Figure:**
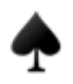


## Figures and Tables

**Table 1:** Learner-Reported Improvements by Skill Domain

Skill Domain	Learners Who Hoped to Improve	Learners Reporting Improvement	Improvement Rate (%)
Airway Management	15	11	73.3
Diagnostic Skills	19	10	52.6
Team-Based Critical Decision-Making	18	15	83.3
Communication Under Pressure	24	14	58.3
Rapid Response and Emergency Intervention	21	16	76.2
Electrocardiogram Interpretation	28	26	92.9
Leadership in High-Stress Situations	22	7	31.8

**Table 2:** Faculty Observations of Effective Skills Taught

Skill Observed as Effectively Taught	Faculty Endorsing (%)
Electrocardiogram Interpretation	100
Diagnostic Skills	83
Team-Based Critical Decision Making	83
Communication Under Pressure	83
Rapid Response and Emergency Intervention	83
Airway Management	67
Leadership in High-Stress Situations	67

**Figure:**
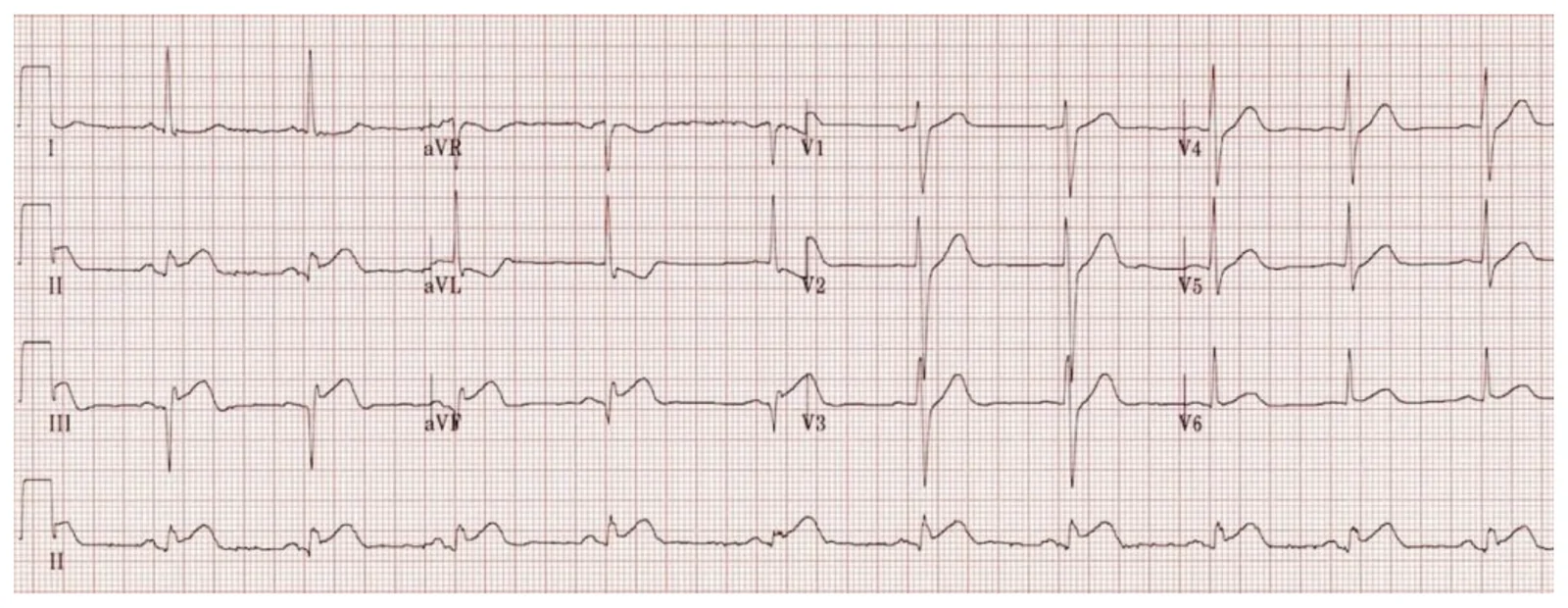
Burns E, Buttner R. Posterior Myocardial Infarction. In: Life in the Fast Lane. Accessed 23 May 2026. CC BY-NC-SA 4.0. https://litfl.com/posterior-myocardial-infarction-ecg-library/

**Figure:**
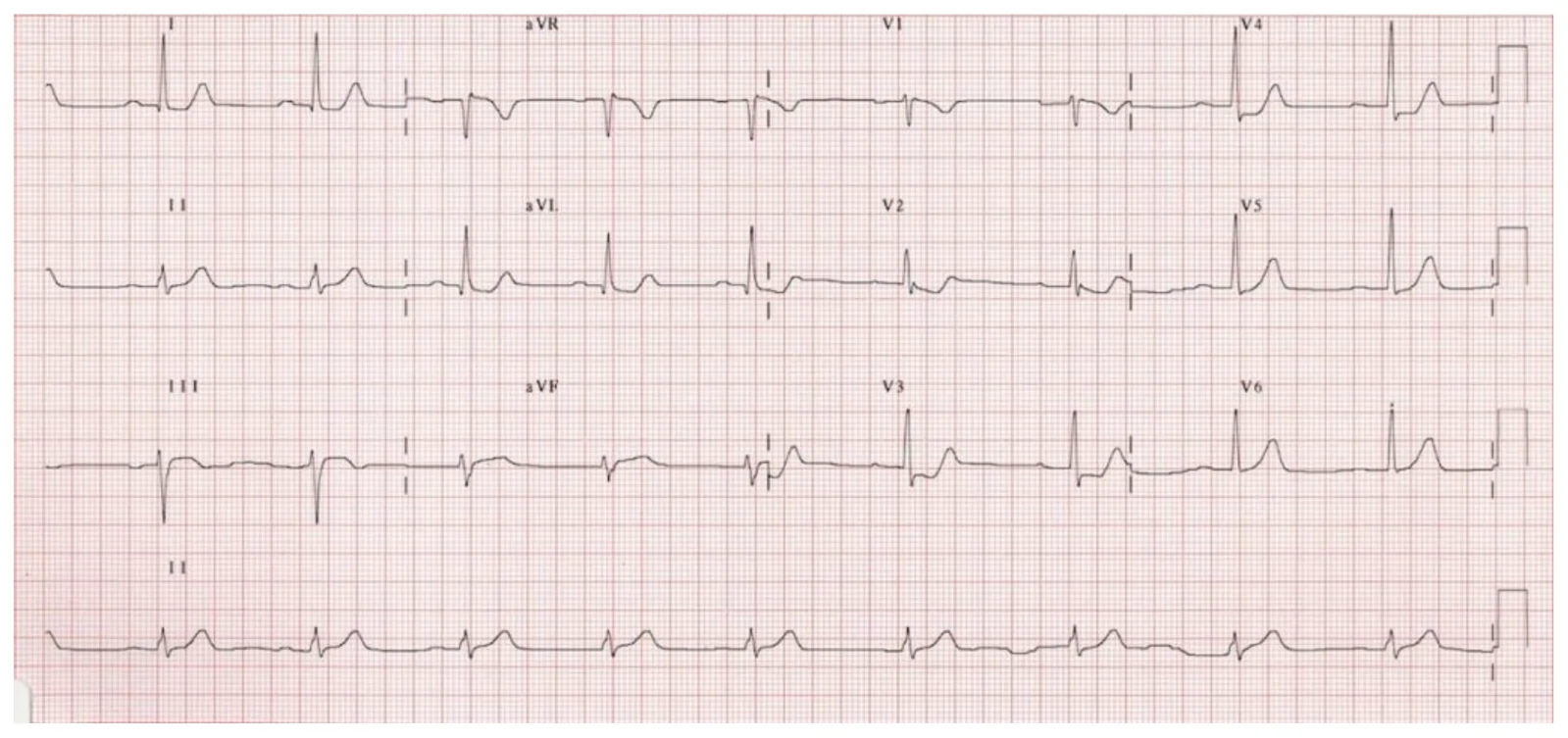
Burns E, Buttner R. Inferior STEMI. In: Life in the Fast Lane. Accessed 23 May 2026. CC BY-NC-SA 4.0. https://litfl.com/inferior-stemi-ecg-library/

**Figure:**
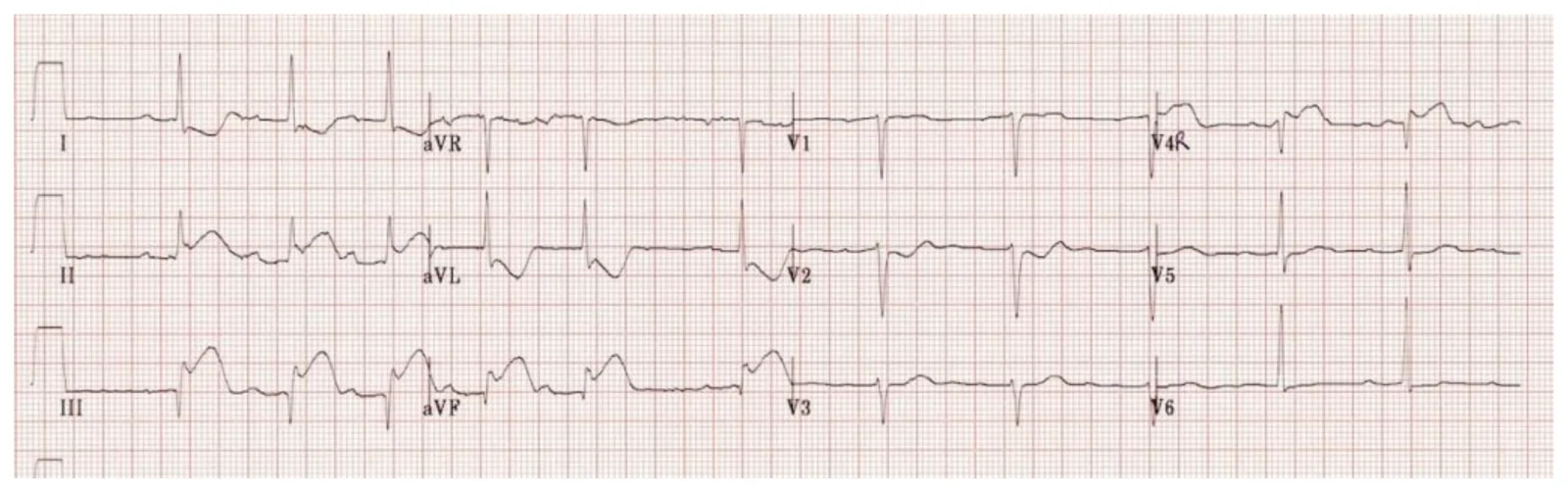
Burns E, Buttner R. Right Ventricular Infarction. In: Life in the Fast Lane. Accessed 23 May 2026. CC BY-NC-SA 4.0. https://litfl.com/right-ventricular-infarction-ecg-library/

**Figure:**
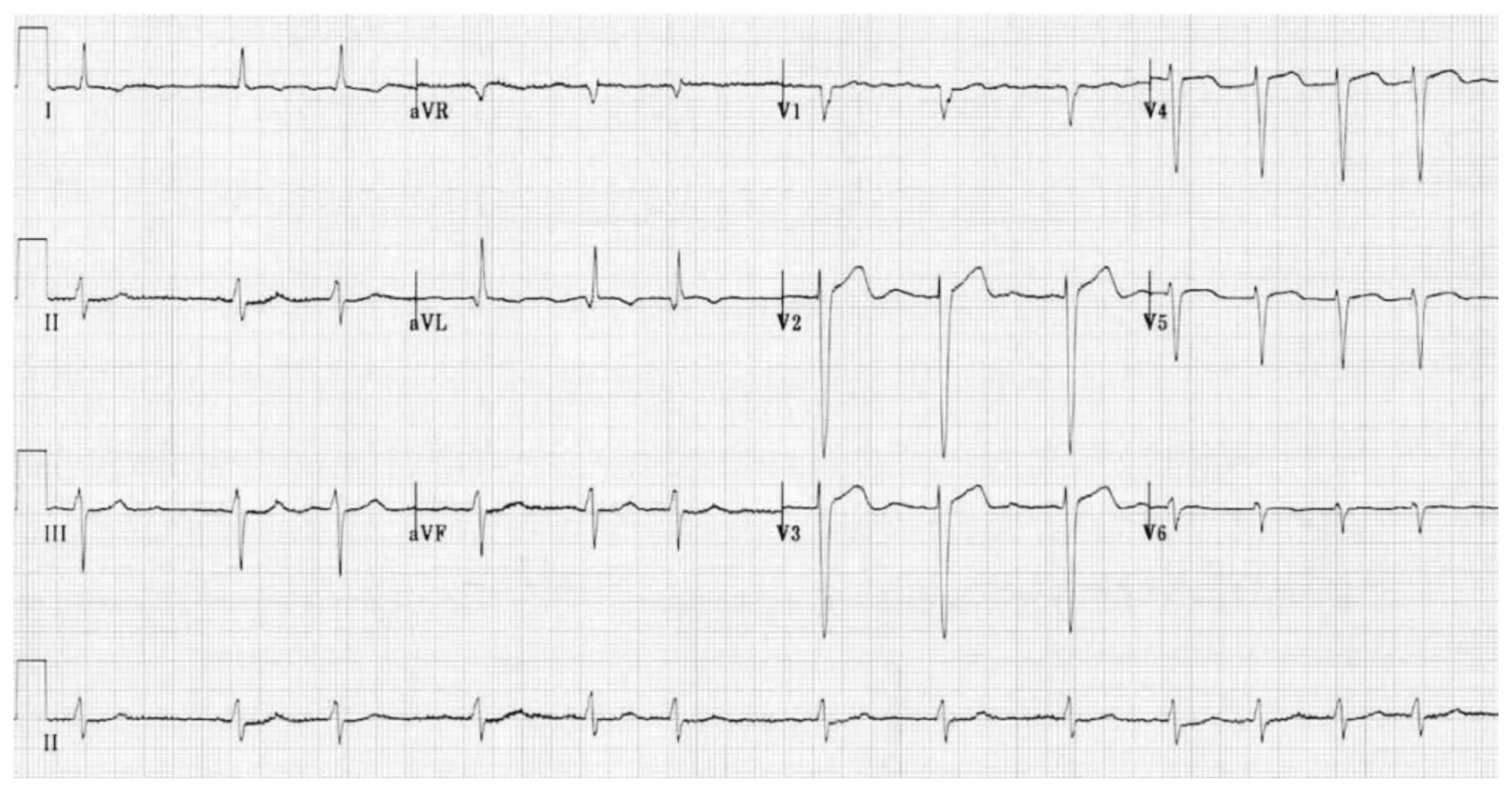
Burns E, Buttner R. Atrial Fibrillation. In: Life in the Fast Lane. Accessed 23 May 2026. CC BYNC-SA 4.0. https://litfl.com/atrial-fibrillation-ecg-library/

**Figure:**
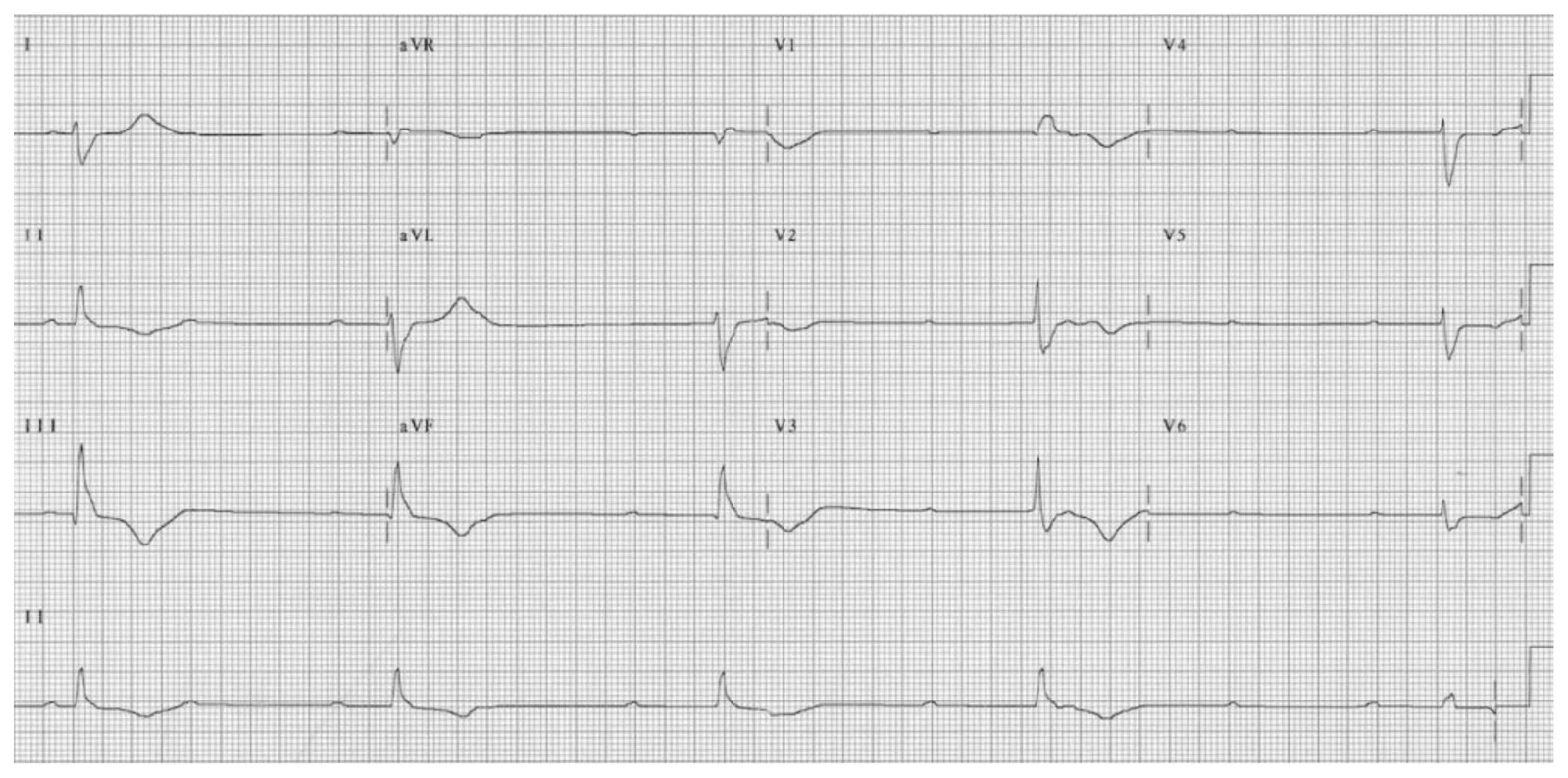
Larkin J, Buttner R. AV block: 3^rd^ degree (complete heart block). In: Life in the Fast Lane. Accessed 23 May 2026. CC BY-NC-SA 4.0. https://litfl.com/av-block-3rd-degree-complete-heart-block/

**Figure:**
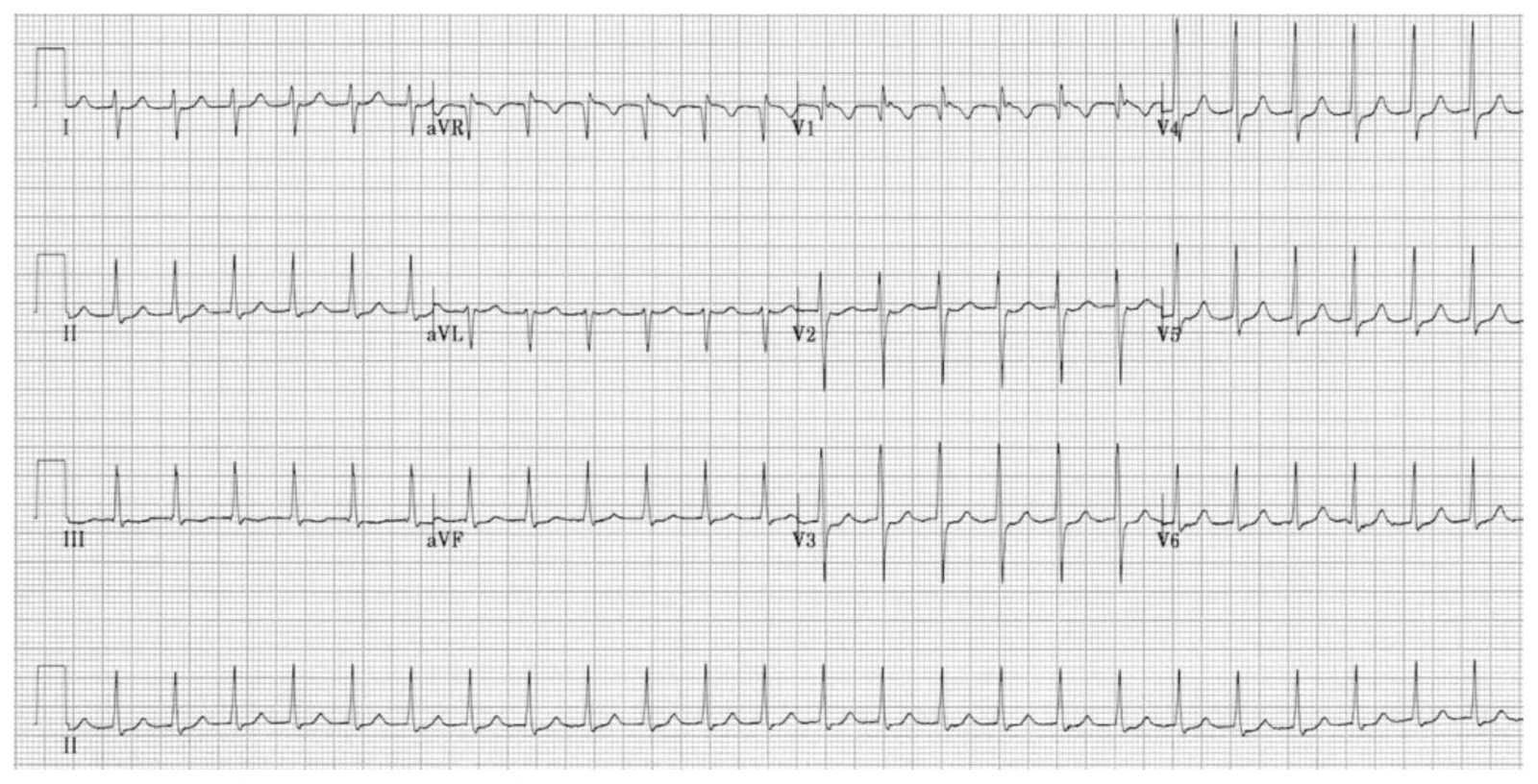
Burns E, Buttner R. Supraventricular Tachycardia. In: Life in the Fast Lane. Accessed 23 May 2026. CC BY-NC-SA 4.0. https://litfl.com/supraventricular-tachycardia-svt-ecg-library/

**Figure:**
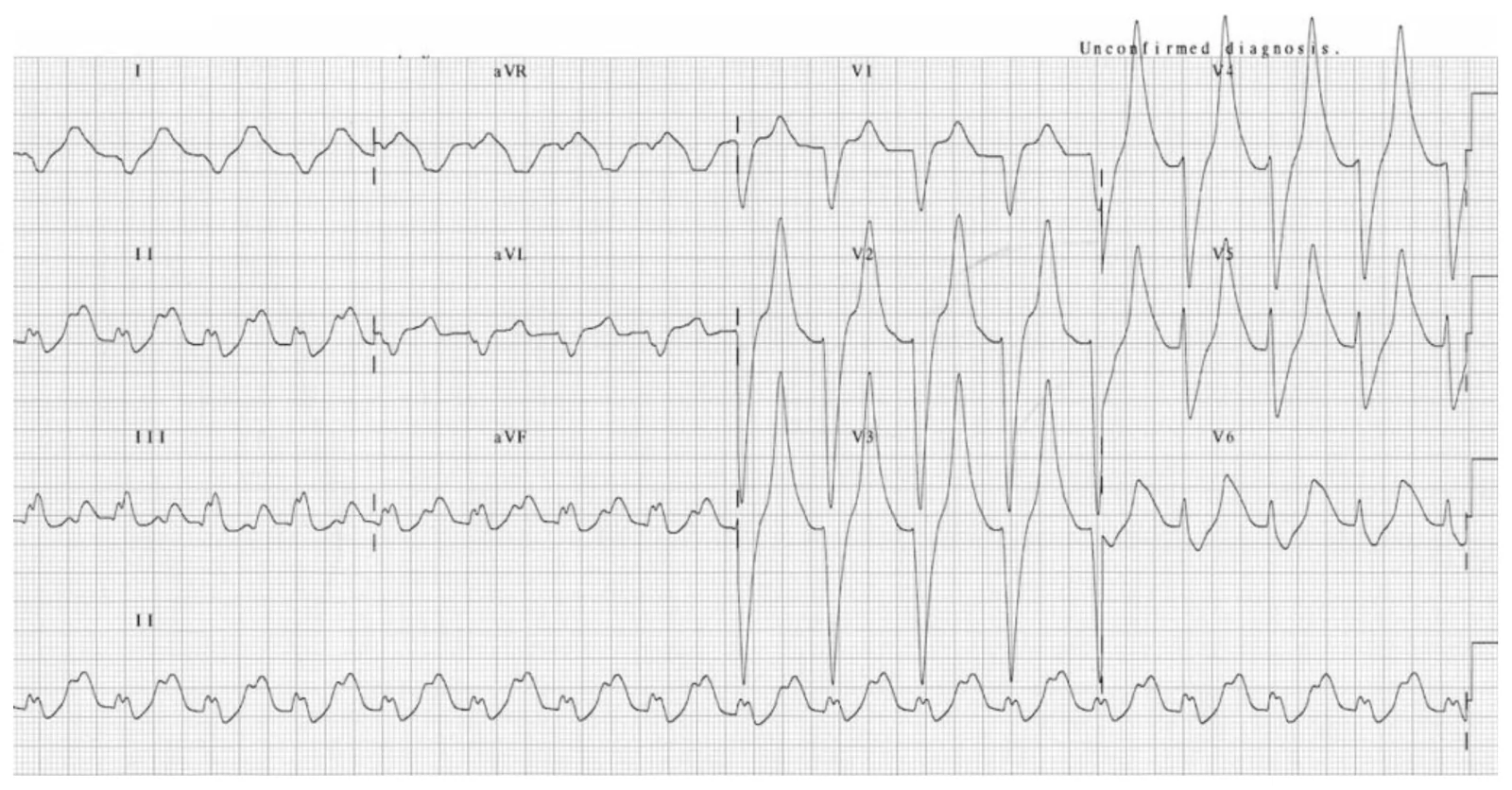
Burns E, Buttner R. Hyperkalaemia. In: Life in the Fast Lane. Accessed 23 May 2026. CC BY-NC-SA 4.0. https://litfl.com/hyperkalaemia-ecg-library/

**Figure:**
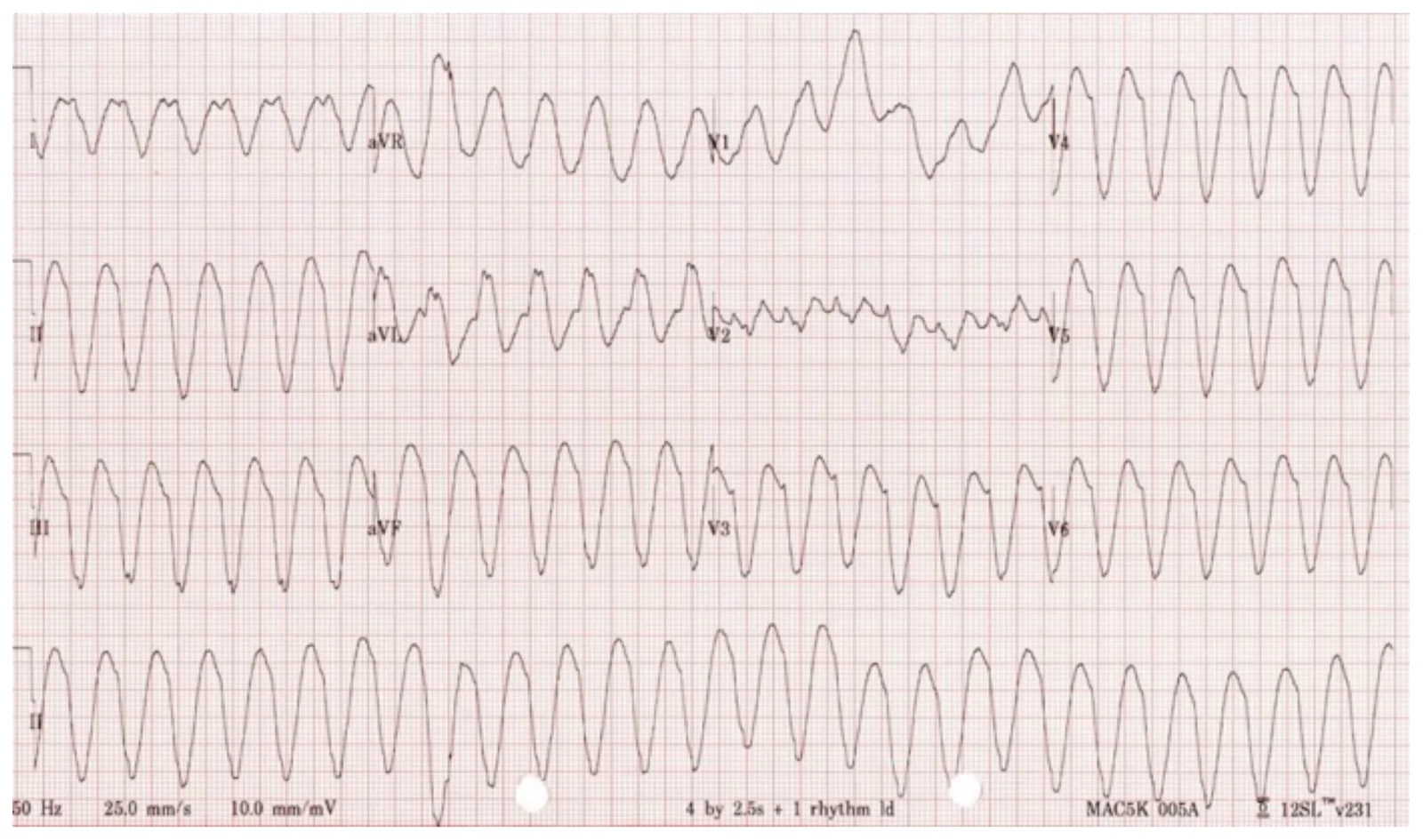
Burns E, Buttner R. Ventricular Tachycardia – Monomorphic VT. In: Life in the Fast Lane. Accessed 23 May 2026. CC BY-NC-SA 4.0. https://litfl.com/ventricular-tachycardia-monomorphic-ecg-library/

**Figure:**
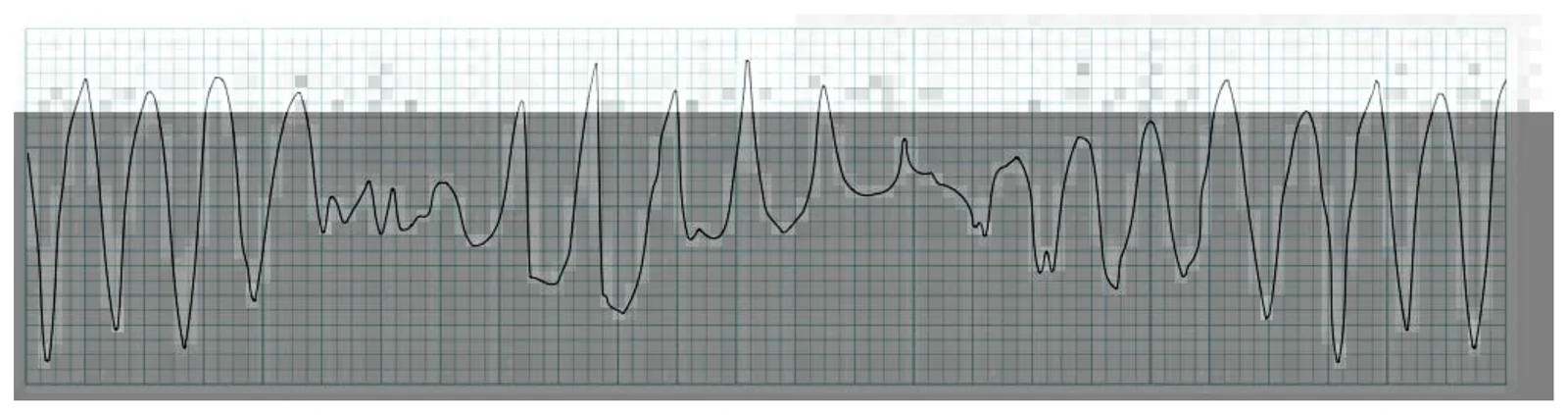
Burns E, Buttner R. Polymorphic VT and Torsades de Pointes (TdP). In: Life in the Fast Lane. Accessed 23 May 2026. CC BY-NC-SA 4.0. https://litfl.com/polymorphic-vt-and-torsades-de-pointes-tdp/

**Figure:**
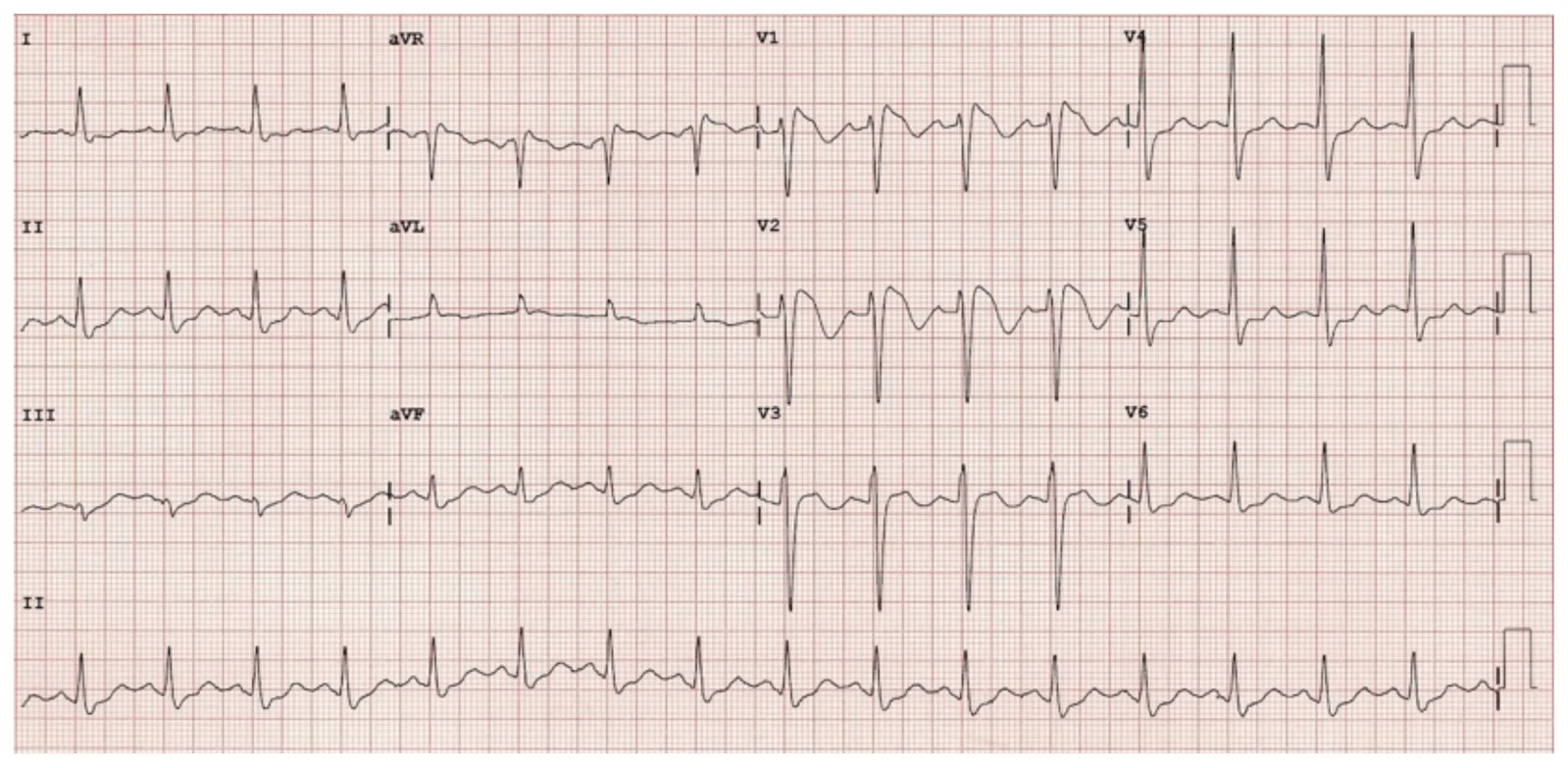
Larkin J, Cadogan M, Buttner R. Brugada Syndrome. In: Life in the Fast Lane. Accessed 23 May 2026. CC BY-NC-SA 4.0. https://litfl.com/brugada-syndrome-ecg-library/

**Figure:**
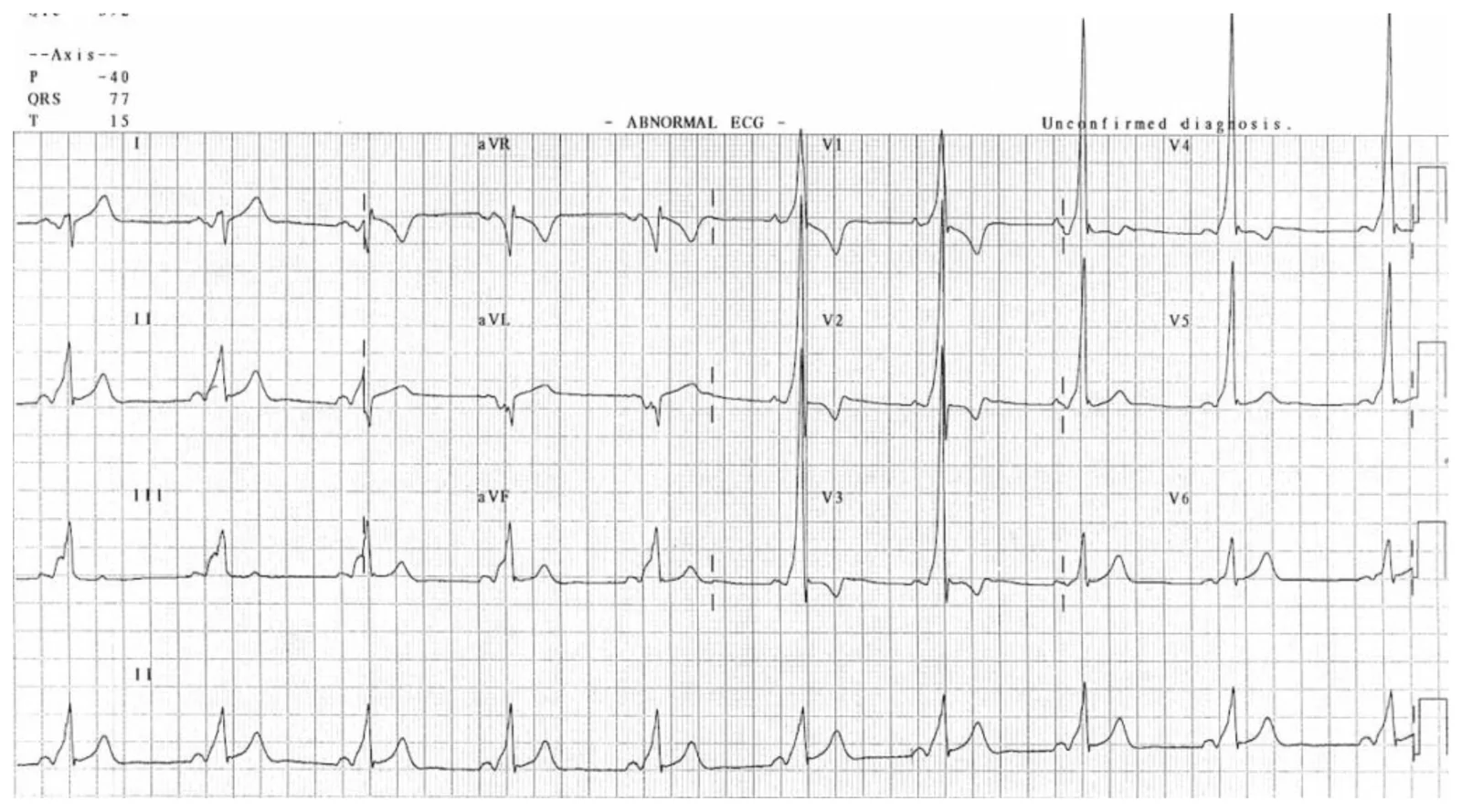
Burns E, Buttner R. Pre-excitation syndromes. In: life in the Fast Lane. Accessed 23 May 2026. CC BY-NC-SA 4.0. https://litfl.com/pre-excitation-syndromes-ecg-library/

**Figure:**
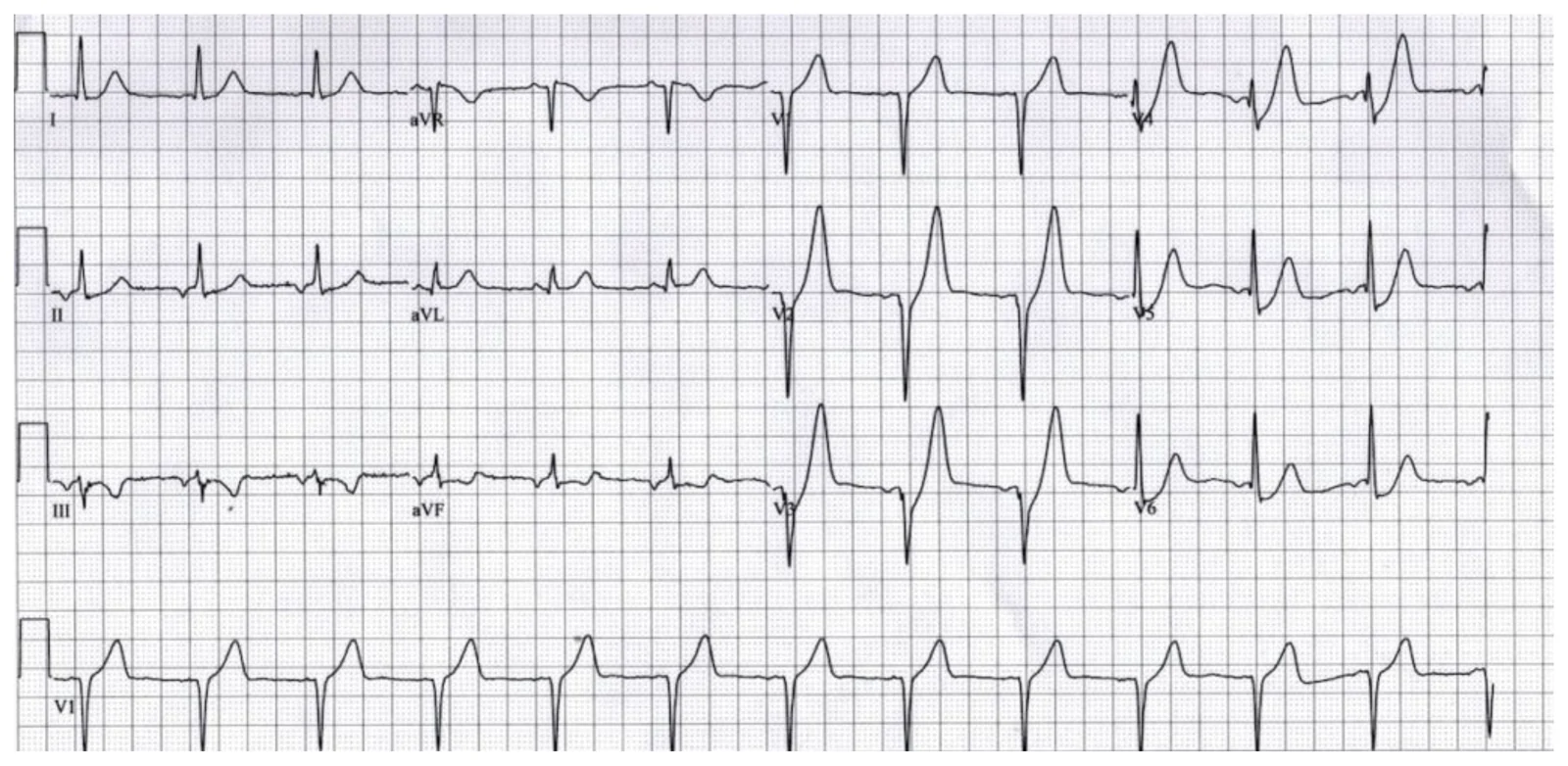
Cadogan M, Buttner R. De Winter T Wave. In: Life in the Fast Lane. Accessed 23 May 2026. CC BY-NC-SA 4.0. https://litfl.com/de-winter-t-wave/

**Figure:**
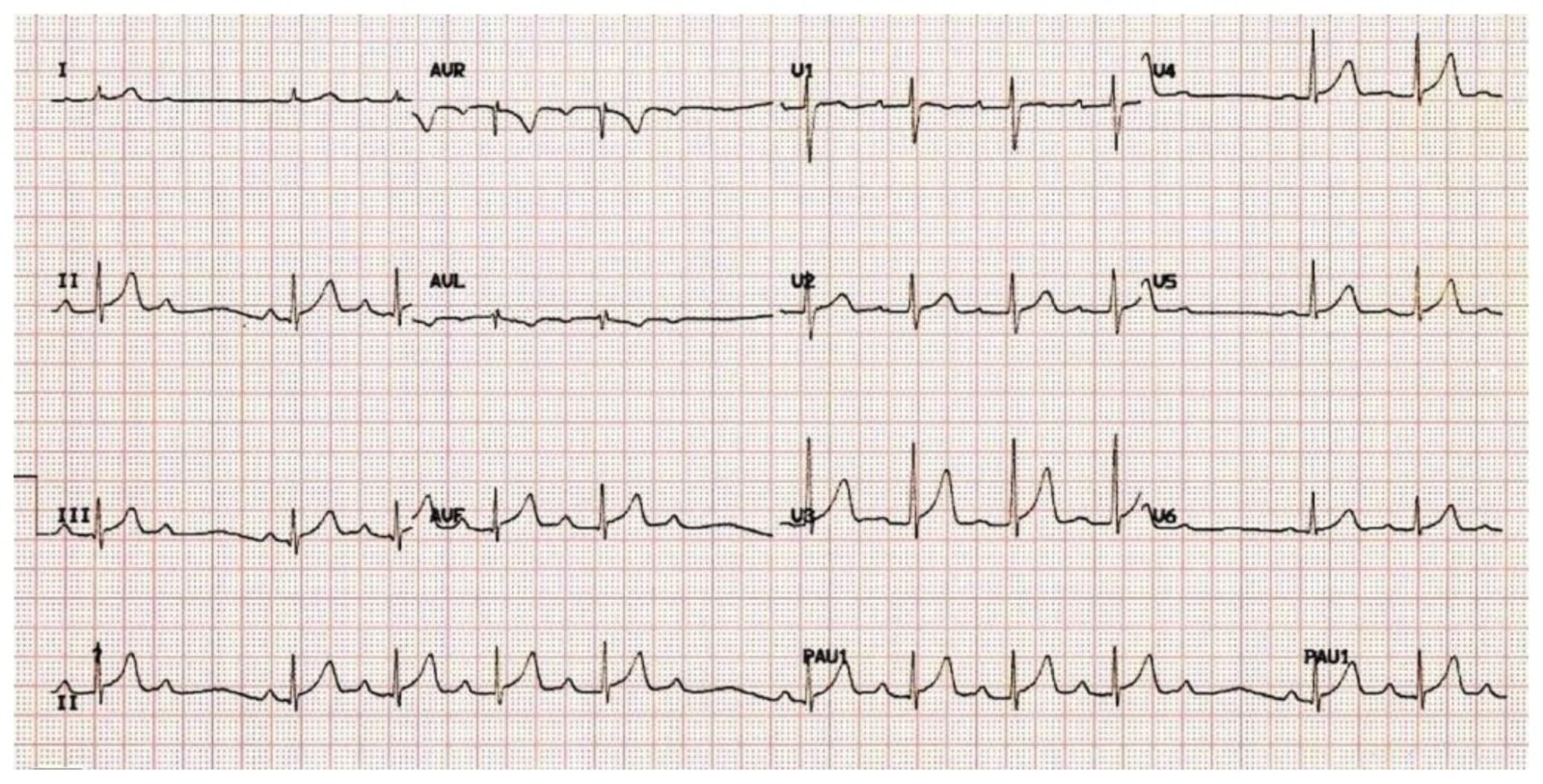
Burns E, Buttner R. AV Block: 2^nd^ degree, Mobitz I (Wenckebach Phenomenon). In: Life in the Fast Lane. Accessed 23 May 2026. CC BY-NC-SA 4.0. https://litfl.com/av-block-2nd-degree-mobitz-i-wencke-bach-phenomenon/

**Figure:**
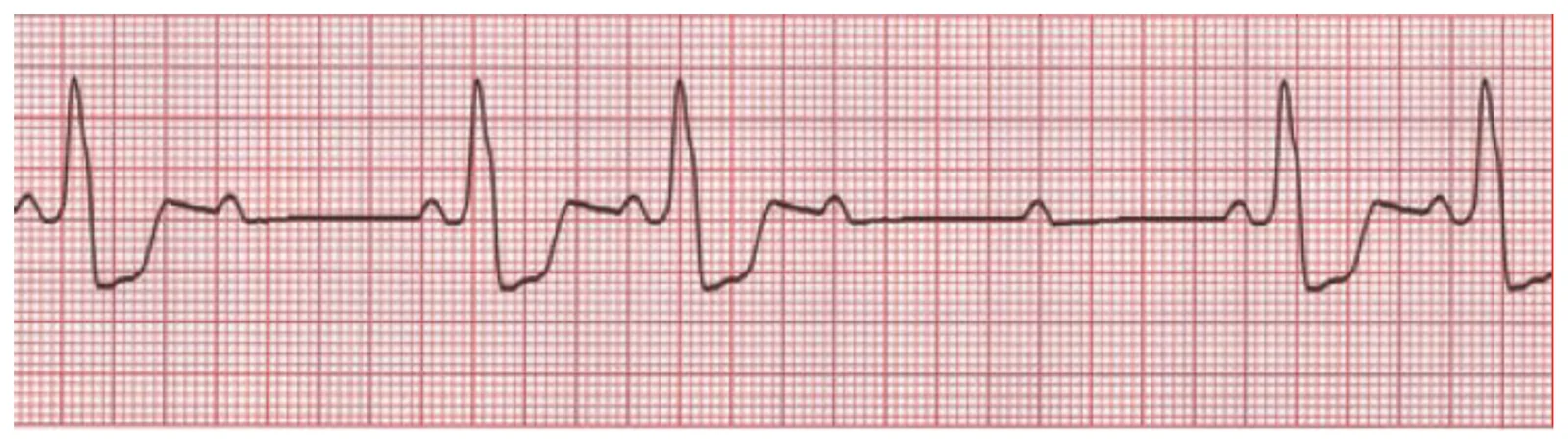
Burns E, Buttner R. AV Block: 2^nd^ degree, Mobitz II (Hay block). In: Life in the Fast Lane. Accessed 23 May 2026. CC BY-NC-SA 4.0. https://litfl.com/av-block-2nd-degree-mobitz-ii-hay-block/

**Figure:**
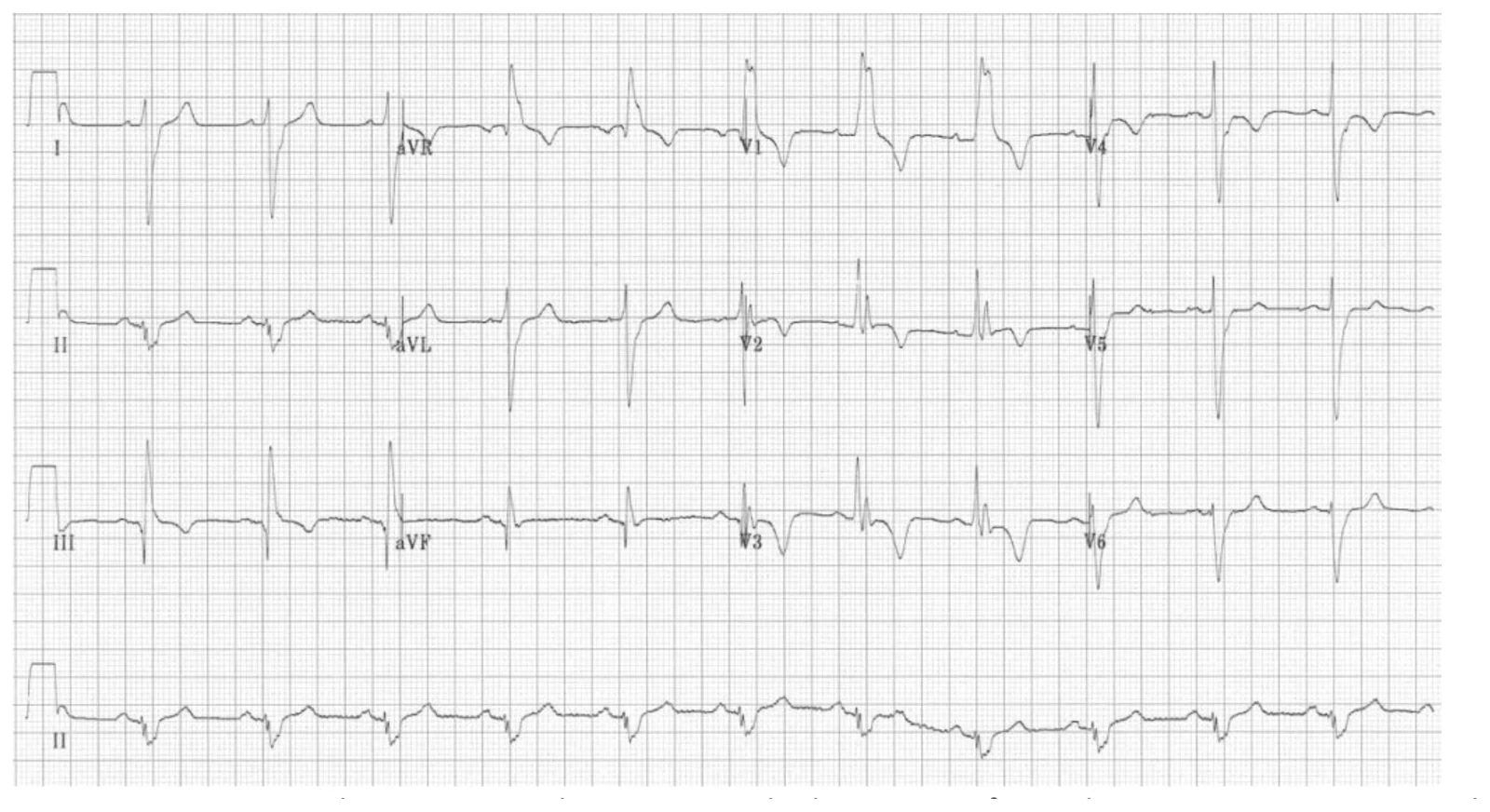
Burns E, Buttner R. ECG changes in Pulmonary Embolism. In: Life in the Fast Lane. Accessed 23 May 2026. CC BY-NC-SA 4.0. https://litfl.com/ecg-changes-in-pulmonary-embolism/

**Figure:**
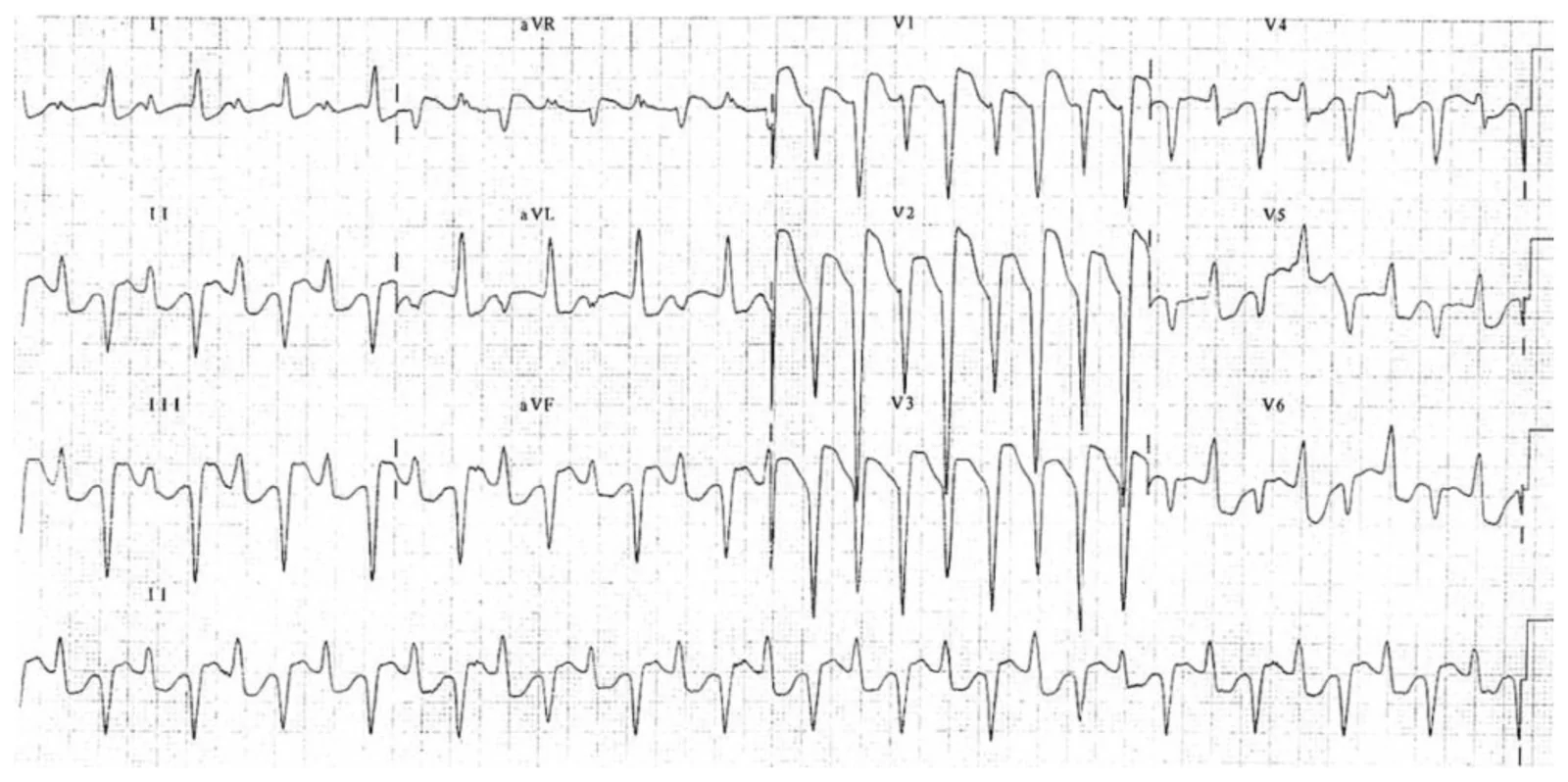
Burns E, Buttner R. Bidirectional Ventricular Tachycardia (BVT). In: Life in the Fast Lane. Accessed 23 May 2026. CC BY-NC-SA 4.0. https://litfl.com/bidirectional-ventricular-tachycardia-bvt-ecg-library/

**Figure:**
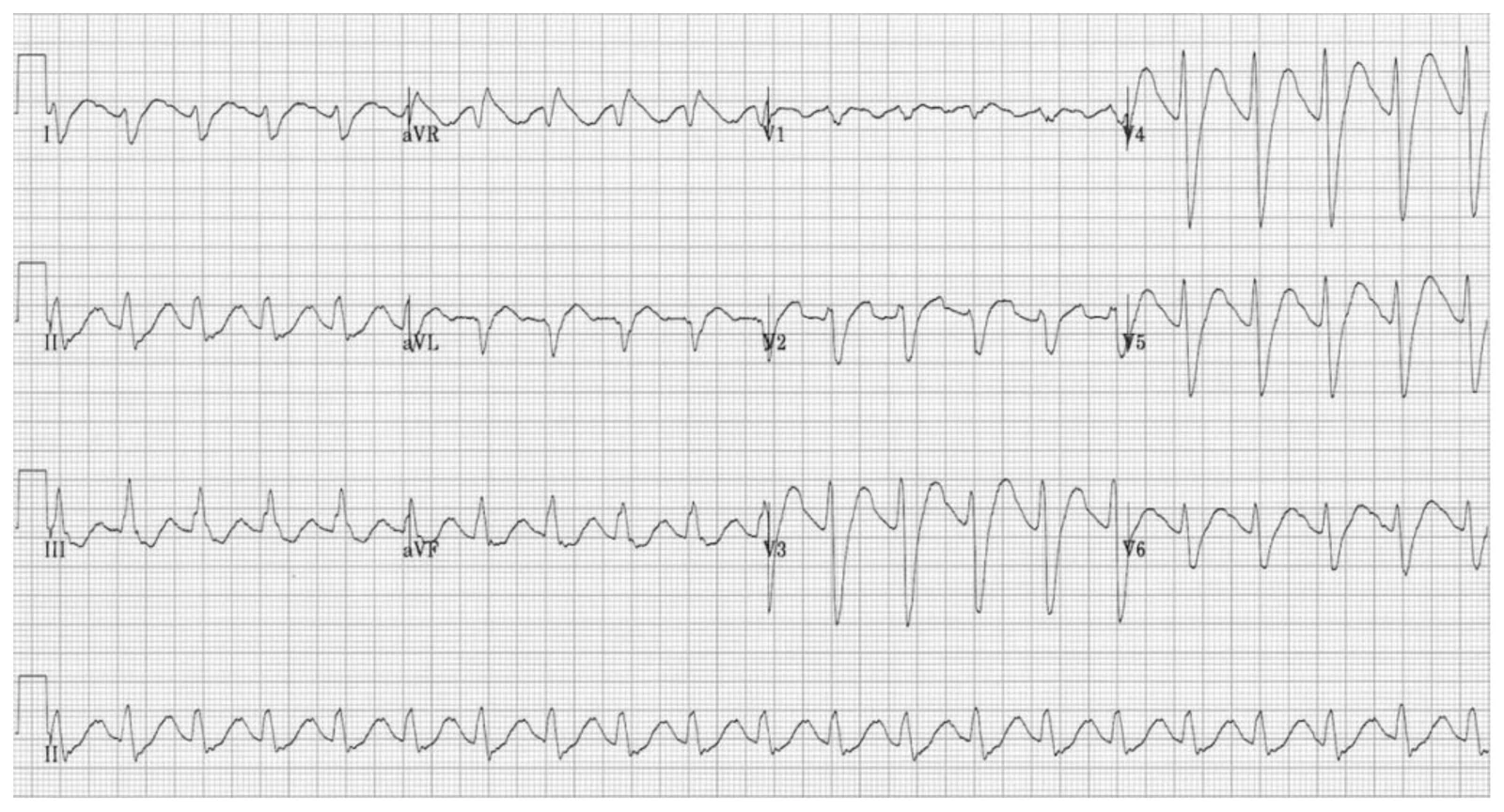
Burns E, Buttner R. Tricyclic Overdose. In: Life in the Fast Lane. Accessed 23 May 2026. CC BY-NC-SA 4.0. https://litfl.com/tricyclic-overdose-sodium-channel-blocker-toxicity/

**Figure:**
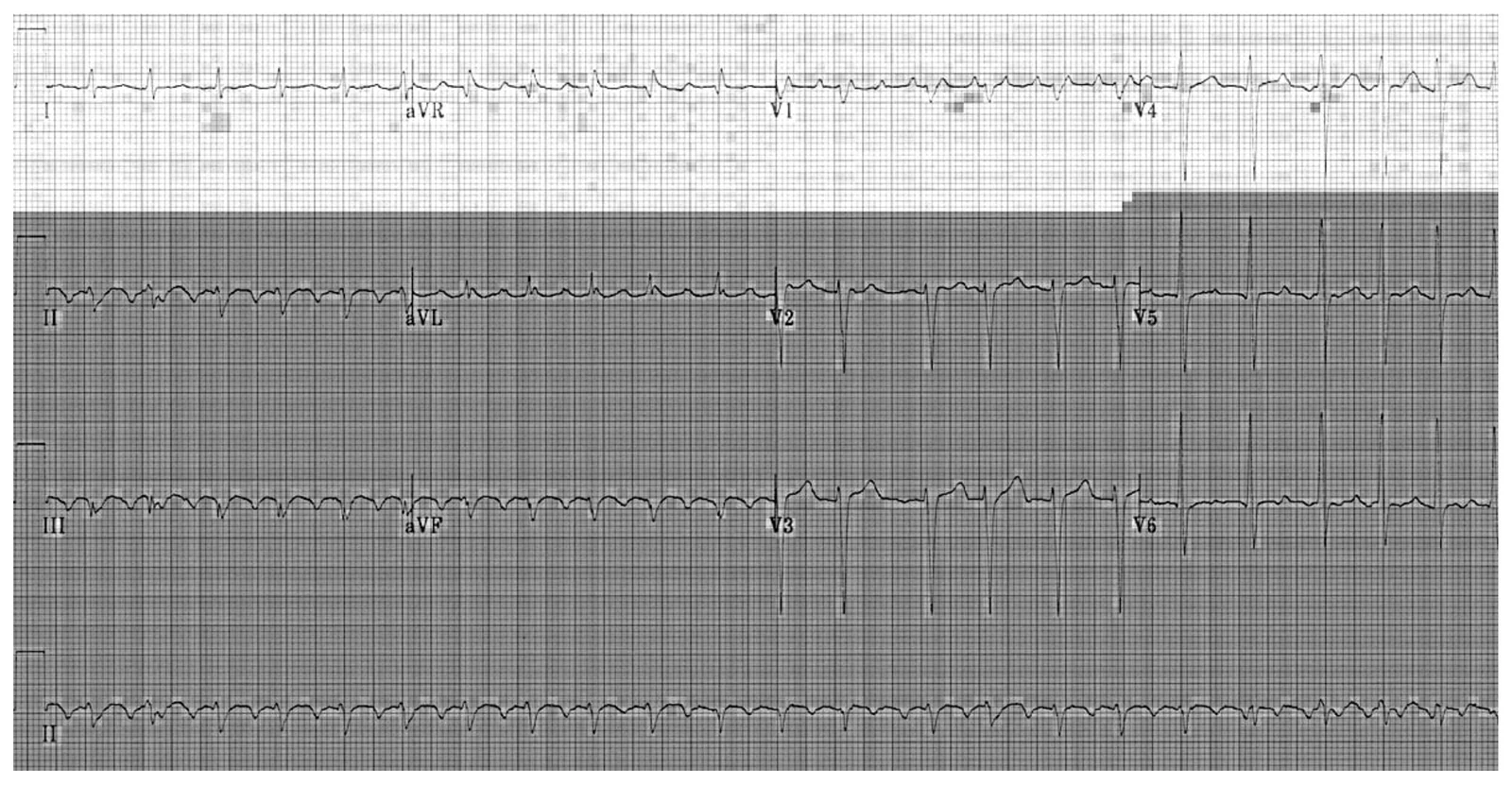
Burns E, Buttner R. Atrial Flutter. In: Life in the Fast Lane. Accessed 23 May 2026. CC BY-NC-SA 4.0. https://litfl.com/atrial-flutter-ecg-library/

## SMALL GROUPS LEARNING MATERIALS

### Small Group Application Exercise (sGAE)

Divide your learners equally into six groups. Have six “casino” tables set up, with one facilitator per table. Based on anticipated attendance, learners can be pre-assigned into six groups (one group per table). Groups can be organized by PGY level, incorporating other learners as you see fit. Facilitators should be EM attending physicians with a background in medical education and simulation encouraged.

*Games* ***:***

1. High Stakes Airways
2. Diagnostic Craps
3. Play Your Cards Right
4. Rapid Response Roulette

Please reference Appendix B, Standardized Facilitator Guide, for the in-detail description of each game and a sample schematic layout.

*Sample Schedule*

**PGY-1/PA Fellow Tables:**
8–9 AM: High Stakes Airways 9–10 AM: Diagnostic Craps 10–11 AM: Play Your Cards Right 11–12 PM: Rapid Response Roulette
**PGY-2 Tables:**
8–9 AM: Rapid Response Roulette 9–10 AM: High Stakes Airways 10–11 AM: Diagnostic Craps 11–12 PM: Play Your Cards Right
**PGY-3 Tables:**
8–9 AM: Play Your Cards Right 9–10 AM: Rapid Response Roulette 10–11 AM: High Stakes Airways 11–12 PM: Diagnostic Craps

*Rules*

Use five different colored poker chips. Each color corresponded to a specific monetary value, as follows:

White = $5 Red =$10 Green = $20 Blue = $50 Black = $100

Each player begins the conference with 15 poker chips, 3 of each color, for a total of $555 for each learner. The maximum bet is $125 per game. Learners are asked to wager their chips based on their confidence before answering questions or completing tasks. Correct responses increase chip totals, while incorrect answers reduce them, introducing a gamified element of accountability and simulating high-stakes ED decision-making.

Facilitators track all bets and wagers during each game at their assigned table. Each station should last 55 minutes with five-minute transitions. At the end of each round, the winner receives the total number of chips wagered by all players, allowing them to gain additional chips beyond their original bet. All other participants then forfeit their wagered chips to the round winner. After all of the four games are completed, the learner with the highest total number of poker chips at each table is declared the table winner. Faculty can provide feedback and debriefing at each station. Standardized facilitator guides (Appendix B) should be distributed prior to the event to ensure instructional consistency.

The physical learning environment should be kept consistent across groups: all sessions run simultaneously with identical time allocations, standardized materials, and facilitator training. A lead EM faculty member is recommended to supervise the entire event, maintain time, and make sure all tables are progressing through the games accordingly.

**Topics:**

- Gamification in medical education
- EM core concepts
- Case-based simulation
- Procedural skills (e.g., airway management)
- Diagnostic decision-making
- Evidence-based medicine (EBM) trivia
- Rapid clinical decision-making

**Appendices:**

- Appendix A: Surveys
- Appendix B: Standardized Facilitator Guide
- sGAE Questions
- sGAE Answers

## Appendix A Surveys

### Pre-Conference Survey

1. **Participant Information**
  - Role:
    ○ EM Resident: PGY-1 PGY-2 PGY-3
    ○ PA Fellow
    ○ Medical Student
    ○ Other (Please specify): _______________
2. **What is your current level of experience with airway management in the ED?**
  - Novice (I have limited experience)
  - Intermediate (I have some experience, but need further practice)
  - Advanced (I am comfortable and confident in managing airways)
  - Expert (I regularly teach others airway management)
3. **How confident are you in making rapid diagnostic decisions in emergency settings (e.g., diagnosing critical conditions such as cardiac arrest or sepsis)?**
  - Not confident
  - Somewhat confident
  - Confident
  - Very confident
4. **How often do you encounter situations requiring rapid responses in the ED? Examples include cardiac arrests, severe traumas, or respiratory failures.**
  - Rarely
  - Occasionally
  - Frequently
  - Very frequently
5. **How confident are you in interpreting an EKG during an emergency situation?**
  - Not confident
  - Somewhat confident
  - Confident
  - Very confident
6. **What do you hope to learn or improve upon by participating in today’s conference?** (Select all that apply)
  - Airway management
  - Diagnostic skills
  - Team-based critical decision making
  - Communication under pressure
  - Rapid response and emergency intervention
  - Electrocardiogram (EKG) interpretation
  - Leadership in high-stress situations
7. **On a scale of 1 to 5, how familiar are you with using games or simulations as a learning tool? (1 = Not familiar, 5 = Extremely familiar).**
  - 1 (Not familiar)
  - 2 (Somewhat familiar)
  - 3 (Moderately familiar)
  - 4 (Very familiar)
  - 5 (Extremely familiar)

### Post-Conference Survey

1. **Participant Information**
  - Role:
    ○ EM Resident: PGY-1 PGY-2 PGY-3
    ○ PA Fellow
    ○ Medical Student
    ○ Other (Please specify): _______________
2. **Rate the educational value of the following activities (Scale: 1 = Not valuable, 5 = Extremely valuable)**
  - **High Stakes Airways (airway management game):**
    **Table:**
    1	2	3	4	5
  - **Diagnostic Craps (diagnostic decision-making game):**
    **Table:**
    1	2	3	4	5
  - **Play Your Cards Right (clinical scenario game):**
    **Table:**
    1	2	3	4	5
  - **Rapid Response Roulette (rapid response simulation):**
    **Table:**
    1	2	3	4	5
3. **To what extent did the games improve your ability to manage high-pressure clinical situations in the ED? Examples include severe traumas or respiratory failures.**
  - Not at all
  - A little
  - Somewhat
  - Quite a bit
  - Definitely
4. **Which of the following skills do you feel you improved upon during the conference? (Select all that apply)**
  - Airway management
  - Diagnostic skills
  - Team-based critical decision-making
  - Communication under pressure
  - Rapid response and emergency intervention
  - Electrocardiogram (EKG) interpretation
  - Leadership in high-stress situations
5. **How likely are you to apply the skills learned from the conference to your clinical practice in the future?**
  - Not likely
  - Somewhat likely
  - Likely
  - Very likely
6. **What did you enjoy most about the conference activities?**
  - Interactive nature of the games
  - Learning through simulation
  - The team-based approach
  - Competition aspect
  - Other: _______________
7. **Do you prefer this type of conference over a didactic conference?**
  - **Yes**
  - **No**
8. **Would you recommend this type of conference to your peers?**
  - Yes
  - No
  - Maybe
9. **How confident do you feel in managing high-stakes situations in the ED after attending this conference? Examples include leading a team during cardiac arrest or making rapid diagnostic decisions.**
  - Not at all
  - A little
  - Somewhat
  - Quite a bit
  - Definitely
10. **Any additional comments or feedback?**

### Post-Conference Survey for Instructors

1. **Instructor Information**
  Role:
  - Attending Physician
  - Other (please specify): ________________________
2. **How would you rate the following activities in terms of their effectiveness as teaching tools?** (Scale: 1 = Not effective, 5 = Extremely effective)
  - **High Stakes Airways** (airway management game):
    **Table:**
    1	2	3	4	5
  - **Diagnostic Craps** (diagnostic decision-making game):
    **Table:**
    1	2	3	4	5
  - **Play Your Cards Right** (clinical scenario game):
    **Table:**
    1	2	3	4	5
  - **Rapid Response Roulette** (rapid response simulation):
    **Table:**
    1	2	3	4	5
3. **To what extent did the games engage learners and enhance their skills in managing high-pressure clinical situations? Examples include scenarios requiring urgent decision-making or leadership during emergencies.**
  - Not at all
  - A little
  - Somewhat
  - Quite a bit
  - Definitely
4. **Which skills did you observe as being most effectively taught through the conference activities? (Select all that apply)**
  - Airway management
  - Diagnostic skills
  - Team-based critical decision-making
  - Communication under pressure
  - Rapid response and emergency intervention
  - Electrocardiogram (EKG) interpretation
  - Leadership in high-stress situations
5. **How would you rate the ease of facilitating each activity?** (Scale: 1 = Very difficult, 5 = Very easy)
  - **High Stakes Airways**:
    **Table:**
    1	2	3	4	5
  - **Diagnostic Craps**:
    **Table:**
    1	2	3	4	5
  - **Play Your Cards Right**:
    **Table:**
    1	2	3	4	5
  - **Rapid Response Roulette**:
    **Table:**
    1	2	3	4	5
6. **How likely are you to incorporate similar game-based learning activities in your future teaching? For example, simulations for airway management or diagnostic decision-making.**
  - Not likely
  - Somewhat likely
  - Likely
  - Very likely
7. **What aspects of the conference did you feel were most successful for teaching?**
  - Interactive nature of the games
  - Realistic simulation scenarios
  - Team-based approach
  - Competition aspect
  - Other: _______________
8. **Do you think game-based learning is more effective than traditional didactic methods for teaching certain skills?**
  - Yes
  - No
  - It depends (please explain): ______________________________
9. **What suggestions do you have for improving the conference from a teaching perspective?**
10. **Any additional comments or feedback?**

## Appendix B Standardized Facilitator Guide

Thank you for being a facilitator for this Casino Conference Day. You will be facilitating a station with 6 participants who are a specific PGY year. Four games will be played throughout the course of the day at your station. Each game will be run over the course of one hour: 55 minutes for the game, and please leave 5 minutes for transitions into the next game and scoring.

**Wagering:** Players may wager any amount of chips they choose **before the question is revealed**. They may also choose to place a multiplier on their wager (e.g., no multiplier, double, or triple). If they answer correctly, they receive their original wager back **plus the winnings based on the multiplier they selected**. If they answer incorrectly, they forfeit their entire wager to the dealer.

**Examples:**

- **- No multiplier:** Wager **$50** → Correct answer → Receive **$100** total ($50 original wager + $50 in winnings).
- **- Double multiplier:** Wager **$50** and choose **Double** → Correct answer → Receive **$150** total ($50 original wager + $100 in winnings).
- **-Triple multiplier:** Wager **$50** and choose **Triple** → Correct answer → Receive **$200** total ($50 original wager + $150 in winnings).

GAMES

1. **High Stakes Airways:** Each participant will be given 2 cases (one adult, one pediatric) corresponding to their PGY year. They will place a bet. You will read them the adult case. They will all write down their answers to the questions following the prompt. They should write answers to both questions before proceeding to the hands-on portion. This is done to make sure everyone answers simultaneously. Collect answers and review them with the players. Each player will then have 5 minutes to perform the intubation on the mannequin. They will be timed to see who has everything set up for the airway and intubates correctly with proper techniques (there will be a checklist for each dealer to check off). Then, they will do the same thing for the pediatric case. Players can verbalize technique for certain aspects (BVM, nasal cannula, etc.) to focus on intubation technique on the mannequin. Participants will be selected in a random order to perform the intubation individually. While one participant is completing the procedure, all remaining participants assigned to that station will wait in a nearby room or designated area where they cannot observe the procedure or communicate with the participant performing the intubation. Once a participant has completed the intubation, they will return directly to their assigned casino conference table and will not return to the waiting area. This process will continue until all participants have completed the station, ensuring that each participant performs the procedure independently without observing or discussing the intubation with others beforehand. The winner will be determined using a point-based scoring system. Each correctly answered question is worth **1 point**, each successfully completed item on the intubation checklist is worth **1 point**, and the participant with the fastest intubation time receives **1 additional point**. The participant with the highest total score across all three categories will be declared the winner. In the event of a tie, the participant with the higher intubation checklist score will be declared the winner. If a tie still remains, the faster intubation time will serve as the final tiebreaker.
2. **Diagnostic Craps:** Each participant will place their bet. Then each participant will roll the dice 3 times to answer 3 questions. Do not make them roll it three times at once. Go down the row, one at a time, having each person go a total of three times (alternate participants). Whoever gets the most correct will get to keep their chips and win their bet, while losers will give their waged chips to the dealer. If multiple players get all three questions correct, they each receive their chips back and win their bet. In case a repeat number is rolled, do not tell them if they got the answer correct. Keep score of who gets the questions correct. If they roll the same number, you can ask a question from a different PGY level. A backup question is provided at the end in case of multiple repeated scenarios. Please remember before having anyone answer their question: show them the corresponding ECG and have them identify what the ECG shows (IE Inferior MI because of… , A fib because of ….). The ECG pattern descriptions are provided as a reminder to do this. At the end, you will review the questions and ECG with the players, letting them know who got the answers correct.
3. **Play Your Cards Right:** Participants will place their bets and then be handed approximately 8 cards each (split the deck evenly amongst the players). They will then have to answer the trivia questions associated with the cards they were dealt. Each player will go one at a time until they finish all their cards (alternate participants). Whoever has the most correct responses wins. The winner gets to keep their chips and win their bet amount, while the losers give their bet number of chips to the dealer.
4. **Rapid Response Roulette:** Participants will place their bets on how many scenarios they think they will be able to answer correctly. Participants will take their turn with the roulette wheel (0, 00, 1–36) and will have to answer the question the ball lands on. Each participant will go as many times as possible within the hour but an equal number per person (alternate participants). Keep score of who gets the questions correct. If they land on the same number, they will have to go again to pick a different question they did not answer. If the player misses more than one third of the steps, they do not get the question correct. The winner gets to keep their chips and win their bet amount, while the losers give their bet to the dealer. For this game, players can “steal” another players chips if a player answers their question incorrectly and another player knows the answer.

**RULES:** Each player will get 15 chips (give each player 3 of each amount); $555 total for each person. The maximum bet is $125 per game.

White = $5 Red = $10 Green = $20 Blue = $50 Black = $100

Note:

- All bets are written down at the same time at the start of each game so players cannot tell what other players are betting.
- If a player loses all their money/chips, the dealer will issue a **$50 House Marker** so the player can continue playing. However, their official score immediately becomes **−$50**. Any future winnings must first repay your debt to the dealer before their score becomes positive again.

SCHEDULE:

**PGY-1:**
8–9 AM: HIGH STAKES AIRWAYS 9–10 AM: DIAGNOSTIC CRAPS 10–11 AM: PLAY YOUR CARDS RIGHT 11–12 PM: RAPID RESPONSE ROULETTE
**PGY-2:**
8–9 AM: RAPID RESPONSE ROULETTE 9–10 AM: HIGH STAKES AIRWAYS 10–11 AM:DIAGNOSTIC CRAPS 11–12 PM: PLAY YOUR CARDS RIGHT
**PGY-3:**
8–9 AM: PLAY YOUR CARDS RIGHT 9–10 AM: RAPID RESPONSE ROULETTE 10–11 AM: HIGH STAKES AIRWAYS 11–12 PM: DIAGNOSTIC CRAPS

**Materials:** Two tables will be playing the same game at the same time.

- (2) decks of Standard Playing Cards (excluding Jokers)
- (2) Roulette Wheels
- (4) Dice
- ~600 Poker Chips
- Simulation equipment: T-piece (2), Wayne pneumothorax kit (2), Adult Intubation Mannequin (2), Pediatric intubation Mannequin (2), Needle Cricothyrotomy set up (2)
- Airway equipment (2): Miller and Mac blade, ET tube and stylet, syringe, bougie, BVM, LMA, nasal cannula, suction, end-tidal CO2

**Schematic Layout for the day:**

**Scoring Card:**

**Table:**

	High Stakes Airways	Diagnostic Craps	Play Your Cards Right	Rapid Response Roulette
Name:				
Name:				
Name:				
Name:				
Name:				
Name:				

### sGAE Questions

#### High Stakes Airways

**PGY-1: Adult Case: Trauma with Suspected C-Spine Injury**

**Case:** A 45-year-old presents after a motor vehicle collision. The patient is unconscious (GCS 7) with shallow respirations and suspected cervical spine injury.

- **Prompt:**
  ○ What medication(s) would you use for intubation? (specify dose and route)
  ○ How does the suspected C-spine injury influence your intubation?

**PGY-1 Pediatric: Epiglottitis in a 4-Year-Old**

**Case:** A 4-year-old child presents with stridor, drooling, and sitting in a tripod position. Oxygen saturation is 89% on room air.

- **Prompt:**
  ○ What medication would you use to sedate and paralyze the patient for emergent intubation? (specify dose and route)
  ○ What pre-intubation strategies and adjuncts should you consider to optimize the airway?

**PGY-2 Adult: GI Bleed with Aspiration Risk**

**Case:** A 58-year-old with a history of liver cirrhosis presents with hematemesis and altered mental status. They are hypoxic with active vomiting.

- **Prompt:**
  ○ What medications would you use for rapid sequence intubation (RSI)? (specify dose and route)
  ○ How does the aspiration risk modify your approach to induction and paralytics?

**PGY-2 Pediatric: Drowning in a 10-Year-Old**

A 10-year-old is brought in after submersion. They are hypoxic with frothy pink sputum and absent respiratory effort.

- **Prompt:**
  ○ What are your choices for RSI medications, considering the hypoxic state? (specify dose and route)
  ○ How would you prepare for potential complications post-intubation?

**PGY-3 Adult: Asthma Exacerbation with Impending Respiratory Failure**

**Case:** A 35-year-old with a history of asthma presents with severe respiratory distress despite three albuterol/ipratropium nebulizations and 2 mg/kg IV methylprednisolone. RR 50, O2 sat 88%, and altered mental status.

1. **Prompt:**
  ○ What medications would you use for intubation? (specify dose and route)
  ○ How would you modify your ventilator settings (tidal volume, respiratory rate) for this patient?

**PGY-3: Foreign Body Aspiration in a 6-Year-Old**

**Case:** A 6-year-old presents with sudden onset stridor and unilateral wheezing after eating peanuts. Oxygen saturation is 92% on 6 L nasal cannula.

- **Prompt:**
  ○ How does the presence of a foreign body change your approach to intubation medications?
  ○ What adjuncts or alternative approaches might you prepare?

#### Diagnostic Craps

**If they roll a total of “2”: STEMI**

“You’re handed this EKG in triage. What would you like to do next?” (Identify STEMI and location) and anything you may consider with this EKG (i.e., vital signs, certain symptoms, which vessel is involved).

- **PGY-1 EKG: Inferior STEMI**
- **PGY-2 EKG: Posterior STEMI**
- **PGY-3 EKG: Right Ventricular STEMI**
- ***If “2” is rolled again:***
  ○ What are the initial medications and interventions for this patient? (show EKG per year)
  ○ The patient develops hypotension post-MI. What are possible causes, and how would you manage them?

**If they roll a total of “3”: Atrial Fibrillation**

**EKG Pattern:** Irregularly irregular rhythm with no distinct P waves.

- **PGY1 Question:** What is this EKG?
- **PGY2 Question:** What factors guide your decision to use rate vs. rhythm control?
- **PGY3 Question:** The patient is unstable. What would you like to do?

**If they roll a total of “4”: Complete (Third-Degree) Heart Block**

**EKG Pattern:** Atrial and ventricular rhythms are dissociated.

- **PGY-1 Question:** What is this EKG? (After they identify the EKG): What finding on this EKG suggests complete heart block?
- **PGY-2 Question:** How would you manage this patient acutely in the ED?
- **PGY-3 Question:** What are the potential causes of this?

**If they roll a total of “5”: Supraventricular Tachycardia (SVT)**

**EKG Pattern:** Narrow QRS complex tachycardia with regular rhythm.

- **PGY-1 Question:** What is this EKG? What are some first-line treatments for a stable patient with this EKG?
- **PGY-2 Question:** What is this EKG? (After they identify the EKG): How do you manage a refractory SVT that doesn’t respond to standard therapy?
- **PGY-3 Question:** How would you manage this in a pediatric patient?

**If they roll a total of “6”: Hyperkalemia**

**EKG Pattern:** Peaked T waves, prolonged PR, wide QRS, sine wave in severe cases.

- **PGY-1 Question:** What does the EKG show and what are you concerned for? (After they identify the EKG): What EKG finding is the earliest indicator of hyperkalemia?
- **PGY-2 Question:** What does the EKG show and what are you concerned for? (After they identify the EKG): What medications are used for immediate stabilization of hyperkalemia?
- **PGY-3 Question:** What does the EKG show and what are you concerned for? The patient is refractory to treatment. What are some other advanced options?

**If they roll a total of “7”: Ventricular Tachycardia (VT)**

**EKG Pattern:** Wide QRS tachycardia; monomorphic or Torsades de pointes for polymorphic.

- **PGY-1 Question:** What medications are appropriate for a stable patient with this EKG?
- **PGY-2 Question:** (Show Torsades EKG) What are the steps to manage this?
- **PGY-3 Question:** What does this EKG show? (After they identify EKG): How do you differentiate VT from SVT with aberrancy?

**If they roll a total of “8”: Brugada Syndrome**

**EKG Pattern:** Coved ST elevation in V1–V3 with a right bundle branch block (RBBB) pattern.

- **PGY-1 Question:** What is this EKG? What is the characteristic EKG finding?
- **PGY-2 Question:** How do you manage a patient presenting with syncope and this EKG?
- **PGY-3 Question:** What considerations should be addressed in a patient with this EKG? (If need prompting can also say genetic or familial considerations).

**If they roll a total of “9”: Wolff-Parkinson-White (WPW)**

**EKG Pattern:** Delta waves with a short PR interval and wide QRS.

- **PGY-1 Question:** What does this EKG show? How do you recognize this on EKG?
- **PGY-2 Question:** What does this EKG show? Follow up with: How do you manage a patient with WPW and atrial fibrillation?
- **PGY-3 Question:** What definitive therapies are available for a patient with this EKG, and how do you counsel patients on long-term management?

**If they roll a total of “10”: De Winter’s T-Waves**

- **EKG Pattern:** Up-sloping ST-depression in precordial leads with tall, symmetric T waves.
- **PGY-1 Question:** Identify this EKG.
- **PGY-2 Question:** What does this EKG show? Which EKG leads are most likely to show the De Winter pattern, and why?
- **PGY-3 Question:** What are the next steps in management if you identify this pattern on the EKG?

**If they roll a total of “11”: Second-Degree AV Block (Type I and II)**

- **PGY-1 Question:** How do you distinguish between these two types of EKG’s (EKG will show Type I and Type II second-degree AV blocks)?
- **PGY-2 Question:** What are the indications for pacing in these patients? Of these two EKG’s, which one would you pace if the patient had any of those indications?
- **PGY-3 Question:** What do each of these EKG’s show? How do you evaluate for reversible causes of this conduction abnormality?

**If they roll a total of “12”:** Identify what is happening with the EKG and give an example of what can cause this.

**Back Up Question: Can use if they repeat a number they already rolled: Atrial Flutter**

**EKG Pattern:** Sawtooth waves, typically at a 2:1 conduction ratio.

- **PGY-1 Question:** What does this EKG show? Follow up with: How do you differentiate atrial flutter from atrial fibrillation?
- **PGY-2 Question:** What are the risks of anticoagulation in this patient, and how do you decide?
- **PGY-3 Question:** What advanced imaging or testing is needed before cardioversion for a patient with this EKG?

#### Play Your Cards Right

##### HEARTS

**ACE:** What are the first-line treatments for symptomatic or severe hypercalcemia?

**TWO:** What medications should you avoid in tricyclic antidepressants (TCA) OD?

**THREE:** What is the most common cause of subarachnoid hemorrhage?

**FOUR:** What is the antidote for opioid overdose?

**FIVE:** What is the most common cause of acute bacterial meningitis in adults?

**SIX:** What is the treatment (medication, route, and dosage) for an anaphylactic reaction?

**SEVEN:** In a patient with suspected tension pneumothorax, what is the initial management step?

**EIGHT:** In the evaluation and management of a suspected acetaminophen (Tylenol) overdose, when should an acetaminophen (APAP) level be obtained? When is N-acetylcysteine (NAC) indicated, and what criteria guide this decision?

**NINE:** What is the correct order of treatment for a patient presenting with status epilepticus in the emergency department?

**TEN:** Which medication is used to reverse the effects of benzodiazepine overdose?

**JACK:** You have a trauma patient with a traumatic hemothorax. What is an indication for emergent exploratory thoracotomy?

**QUEEN:** Name the unstable C-spine fractures.

**KING:** What is the management for a patient with suspected pulmonary embolism (PE)?

##### DIAMONDS

**ACE:** What is the first-line treatment for acute pancreatitis?

**TWO:** What is the most common cause of small bowel obstruction in adults?

**THREE:** What condition should be considered in a patient with new onset confusion, fever, and headache who has a history of a recent upper respiratory infection?

**FOUR:** What is the most common cause of hypoglycemia in an inpatient setting?

**FIVE:** What is the most common cause of cardiogenic shock?

**SIX:** What is the management for a patient with acute epiglottitis?

**SEVEN:** What is the most common cause of upper GI bleeding?

**EIGHT:** What is the antidote for cyanide poisoning?

**NINE:** What is the most common cause of sudden cardiac death in young athletes?

**TEN:** What is the first-line treatment for acute asthma exacerbation?

**JACK:** What is the management of a patient with suspected acute adrenal crisis?

**QUEEN:** What is the next escalation in care for a bronchiolitis patient whose work of breathing increases?

**KING:** A 5-year-old (18 kg) presents with lethargy and a blood glucose level of 32 mg/dL. What immediate treatment should you give and what is the dose?

##### CLUBS

**ACE:** What is the most common complication of endotracheal intubation?

**TWO:** A 4-year-old presents with intermittent abdominal pain and currant jelly stools. What condition is most likely?

**THREE:** What is the most common cause of acute interstitial pneumonia in immunocompromised patients?

**FOUR:** A 6-year-old with fever and refusal to bear weight has a hip effusion on ultrasound. What is the most likely diagnosis? What criteria will help you assess this (must list)?

**FIVE:** What medication should be started immediately in a newborn with suspected ductal-dependent congenital heart disease and what is the main side effect of this medication?

**SIX:** What is the treatment for calcium channel blocker overdose?

**SEVEN:** In the setting of a suspected salicylate overdose, what is the initial treatment?

**EIGHT:** A 2-year-old with a generalized seizure lasting 8 minutes and fever returns to baseline. What is the likely diagnosis?

**NINE:** A 3-year-old child presents after swallowing a coin. If the foreign body is located in the proximal esophagus but the child is otherwise asymptomatic, how long is it appropriate to observe before intervention is required?

**TEN:** What is the treatment for a patient with rash and fever after recent tick exposure?

**JACK:** What is the most common cause of postpartum hemorrhage (PPH)?

**QUEEN:** What is the most common cause of upper airway obstruction in children under 6 years of age?

**KING:** What are the symptoms of atropine overdose, and how is it treated?

##### SPADES

**ACE:** In a tension pneumothorax, what are the anatomical landmarks for needle decompression?

**TWO: W**hat are the first-line treatments for organophosphate poisoning?

**THREE:** What is the most common ECG finding in a patient with a left ventricular aneurysm following a myocardial infarction?

**FOUR:** What is the treatment of choice for a patient with symptomatic hyperthyroidism (thyroid storm)?

**FIVE:** What is the most common type of shock in a trauma patient with spinal cord injury?

**SIX:** What is the most common complication in a patient with acetaminophen overdose?

**SEVEN:** What is the antidote for methanol poisoning?

**EIGHT:** A 30-year-old male presents with altered mental status, tachycardia, and hyperthermia after ingesting a “designer drug” at a party. What is the most likely diagnosis, and how is it managed?

**NINE:** What is the primary treatment for a patient with acute angle-closure glaucoma?

**TEN:** What is the recommended tidal volume setting for ventilating a patient with acute respiratory distress syndrome (ARDS)?

**JACK:** What is the first-line treatment for a patient with a suspected acute iron overdose?

**QUEEN:** What is the first-line treatment for a patient with carbon monoxide poisoning?

**KING:** What imaging modality is diagnostic and therapeutic for intussusception?

#### Rapid Response Roulette

**If they land on “1”: VF/VT Arrest in a Known CAD Patient**

*Scenario*: A 63-year-old female with a history of coronary artery disease goes into ventricular fibrillation after being brought in for chest pain. Question: What are the next steps?

**If they land on “2”: Asystole Arrest**

*Scenario*: A 70-year-old male found unresponsive with asystole on the monitor. You’ve already intubated. Question: What are the next steps?

**If they land on “3”: Pulseless Electrical Activity (PEA) Arrest**

*Scenario*: A 50-year-old male who arrested with a rhythm of sinus tachycardia but no pulse. Question: What are the next steps?

**If they land on “4”: Hypothermic Cardiac Arrest**

*Scenario*: A 32-year-old male with hypothermic arrest after being found in a snowbank. Question: What are the next steps?

**If they land on “5”: TCA Overdose Cardiac Arrest**

*Scenario*: A 28-year-old female who overdosed on antidepressants with a non-shockable rhythm and no pulse (Show EKG: it will show a prolonged QRS duration and a wide terminal R wave in lead aVR). Question: What are the next steps?

**If they land on “6”: Torsades de Pointes**

*Scenario*: 30-year-old male hx substance use disorder on methadone, presenting after collapse and unresponsiveness. HR 0 (cardiac arrest), irregular, polymorphic QRS complexes on EKG. Question: What are the next steps?

**If they land on “7”: Sepsis in a Diabetic Patient**

*Scenario*: A 60-year-old diabetic male presenting with hypotension, fever, and confusion after a urinary tract infection. Question: What are the next steps?

**If they land on “8”: Neonatal Sepsis**

*Scenario*: A 2-day-old infant with lethargy, poor feeding, and temp of 101.4° F rectally. Question: What are the next steps?

**If they land on “9”: Sepsis in an Immunocompromised Patient**

*Scenario*: A 50-year-old female on chemotherapy, presenting with fever, tachycardia, and hypotension. Question: What are the next steps?

**If they land on “10”: Necrotizing Fasciitis**

*Scenario*: A 55-year-old male presents with severe, rapidly progressing pain, redness, and swelling in his right lower leg. He has a history of diabetes mellitus and recent minor trauma to the leg. On examination, the skin is erythematous, and there are signs of bullae and crepitus. The patient appears febrile and toxic. Question: What are the next steps?

**If they land on “11”: Blunt Chest Trauma with Hemothorax**

*Scenario*: A 30-year-old male involved in a motor vehicle crash, now with hypotension, respiratory distress, and decreased breath sounds on the right. Question: What are the next steps?

**If they land on “12”: Penetrating Abdominal Trauma**

*Scenario*: A 45-year-old male with a stab wound to the left upper quadrant and signs of peritonitis. Question: What are the next steps?

**If they land on “13”: Traumatic Brain Injury (TBI)**

*Scenario*: A 20-year-old male involved in a motorcycle accident with a GCS of 7 and unequal pupils. Question: What are the next steps?

**If they land on “14”: Pelvic Fracture with Shock**

*Scenario*: A 50-year-old male after a high-speed car accident presenting with hypotension and pelvic fractures. Question: What are the next steps?

**If they land on “15”: Trauma with Suspected Compartment Syndrome**

*Scenario*: A 40-year-old male with a fractured tibia and increased pain despite analgesia. Question: What are the next steps?

**If they land on “16”: Burns with Airway Compromise**

*Scenario*: A 22-year-old male with third-degree burns to the face and neck and signs of respiratory distress. Question: What are the next steps?

**If they land on “17”: Crush Injury with Renal Complications**

*Scenario*: A 28-year-old male trapped under debris after an industrial accident presenting with dark urine. Question: What are the next steps?

**If they land on “18”: Acute Ischemic Stroke**

*Scenario*: A 70-year-old male presenting with sudden-onset right-sided weakness, facial droop, and speech difficulty. Question: What are the next steps?

**If they land on “19”: Hemorrhagic Stroke**

*Scenario*: A 65-year-old female with sudden severe headache, vomiting, and altered mental status. CT head shows a large hemorrhage. Question: What are the next steps?

**If they land on “20”: Severe Asthma Exacerbation**

*Scenario*: A 25-year-old female presents with wheezing, increased work of breathing, and a history of asthma. She is using her inhaler without relief. Question: What are the next steps?

**If they land on “21”: Acute Anaphylaxis**

*Scenario*: A 30-year-old male with a history of allergies presents with swelling of the face, difficulty breathing, and hives after eating shellfish. Question: What are the next steps?

**If they land on “22”: Acute Gastrointestinal Bleed**

*Scenario*: A 50-year-old male with a history of alcohol use presents with hematemesis and hypotension. Question: What are the next steps?

**If they land on “23”:** Describe the steps for a SALAD (Suction-Assisted Laryngoscopy and Airway Decontamination) intubation.

**If they land on “24”: Heat Stroke**

*Scenario*: A 22-year-old male marathon runner presents with a core temperature of 104° F (40° C), confusion, and tachycardia. Question: What are the next steps?

**If they land on “25”: Epistaxis**

*Scenario*: A 45-year-old male presents with profuse, uncontrollable nosebleed after a recent head trauma. Question: What are the next steps?

**If they land on “26”: Alcohol Withdrawal**

*Scenario*: A 40-year-old male with a history of chronic alcohol use presents with tremors, agitation, and confusion 24 hours after his last drink. Question: What are the next steps?

**If they land on “27”: Thyroid Storm**

*Scenario:* Patient: 45-year-old female with Graves’ disease, fever (103° F), agitation, confusion, tachycardia (150 bpm), recent URI, non-compliance with antithyroid meds. Vitals: BP 160/90 mmHg, HR 150 bpm, Temp 103° F. Question: What are the next steps?

**If they land on “28”: Needle Cricothyrotomy**

Explain/demonstrate how to do this on the trainer.

**If they land on “29”: DKA (Diabetic Ketoacidosis)**

*Scenario:* A 17-year-old female with diabetes mellitus type 1 (DM1) presents with 2 days of vomiting, abdominal pain, and confusion. Vital signs show HR 120, BP 100/65, RR 28, and SpO2 98% on room air. Labs reveal glucose 580 mg/dL, pH 7.18, HCO3 12 mEq/L, and ketones present in urine and serum. Question: What are the next steps?

**If they land on “30”:** Describe how to place a **Wayne PTX catheter:** Have kit nearby and let them show and explain steps.

**If they land on “31”:** Describe indications and how to do a **lateral canthotomy.**

**If they land on “32”:** Describe the steps to use a **T-piece**. (Use model to demonstrate).

**If they land on “33”: Nursemaid Elbow Reduction**: Please demonstrate and describe the steps.

**If they land on “34”: Myxedema Coma**

*Scenario:* A 65-year-old woman with a history of hypothyroidism presents with confusion, hypothermia (T 34° C), bradycardia (HR 50), hypotension (BP 80/50), and edema. Labs show thyroid stimulating hormone (TSH) 85 μIU/mL and thyroxine (free T4) <0.2 ng/dL. Question: What are the next steps?

**If they land on “35”: Anterior Shoulder Dislocation Reduction**

*Scenario:* A 25-year-old male presents after a fall onto an outstretched arm during a basketball game. His right shoulder is visibly deformed, and he holds it in slight abduction and external rotation. He reports severe pain and inability to move the arm. X-rays confirm an anterior shoulder dislocation with no associated fractures. Demonstrate a technique on a partner.

**If they land on “36”: SBP (Spontaneous Bacterial Peritonitis) and Paracentesis**

*Scenario:* A 52-year-old male with a history of cirrhosis secondary to alcohol use presents with fever, abdominal pain, and confusion. On exam, he has a distended abdomen with shifting dullness. Vital signs show HR 110, BP 95/60, RR 20, and T 38.3° C. Labs reveal elevated WBCs and worsening kidney function. You suspect SBP and decide to perform a diagnostic paracentesis. Describe how to manage SBP and perform a paracentesis.

**If they land on “0”: Lumbar Puncture (LP)**

*Scenario: A 2-week-old infant presents with fever and irritability. You suspect herpes simples virus (HSV) encephalitis and need to perform a lumbar puncture (LP) for cerebrospinal fluid (CSF) analysis.* Describe how to perform a pediatric LP.

**If they land on “00”: Cardiac Tamponade**

*Scenario:* A 58-year-old male with a history of metastatic lung cancer presents with chest pain, dyspnea, and lightheadedness. On exam, he is hypotensive (BP 85/60), tachycardic (HR 120), and has distended neck veins with muffled heart sounds. Bedside ultrasound shows a large pericardial effusion with diastolic collapse of the right ventricle. Describe how to do a pericardiocentesis.

***Extra scenarios in case they land on the same number twice:***

**Septic Joint:** A 55-year-old female presents with a swollen, painful knee joint, fever and decreased range of motion (ROM) of knee. Describe how to perform arthrocentesis on the knee.

**Describe how to perform a central line placement via the subclavian vein.**

**Massive Transfusion Protocol (MTP):** You have a trauma patient that is actively hemorrhaging, BP 60/palp. You decide to activate an MTP. What does this entail?

### sGAE Answers

#### High Stakes Airways

**PGY-1: Adult Case: Trauma with Suspected C-Spine Injury**

- **Expected Answers:** ^**13**^
  ○ Medications:
    ▪ **Sedative**: Etomidate (0.3 mg/kg IV) is often considered the best medication for induction due to its minimal cardiovascular effects and potential to decrease intracranial pressure (ICP), making it a safer choice for patients with potential spinal cord damage.
    ▪ Can consider ketamine, at high doses may increase ICP.
    ▪ **Paralytic**: Rocuronium (1 mg/kg IV) for paralytic.
  ○ Maintain in-line stabilization of the neck to prevent further spinal cord damage, typically achieved through manual in-line stabilization.
  ○ Prepare for a difficult airway backup plan:
    ▪ Video laryngoscopy: Preferred method for many clinicians due to better visualization and less need for neck manipulation.
    ▪ Fiberoptic intubation: May be used in certain situations where awake intubation is possible and considered safer.
    ▪ Bougie: Can be used as an adjunct to facilitate tube placement, especially in difficult airways.

**PGY-1 Pediatric Case: Epiglottitis in a 4-Year-Old**

- **Expected Answers:** ^**14**^
  ○ **Sedative:** Ketamine (1–2 mg/kg IV) for sedation and airway reflex preservation.
  ○ **Paralytic:** Succinylcholine (1–2 mg/kg IV) or rocuronium (1 mg/kg IV).
  ○ **Adjuncts:** Plan for a backup airway with ENT or surgical cricothyrotomy in case of failed intubation. Can also consider taking patient to the operating room for intubation to avoid increasing distress.

**PGY-2 Adult Case: GI Bleed with Aspiration Risk**

- **Expected Answers:** ^**15**^
  ○ **Sedative:** Etomidate (0.2–0.4 mg/kg IV) or ketamine (1–2 mg/kg IV for hemodynamic stability).
  ○ **Paralytic:** Rocuronium (1 mg/kg IV).
  ○ **Key Considerations:** Ensure suction is ready (DuCanto), use a rapid induction technique to minimize aspiration risk, and plan for potential difficult airway due to blood in the oropharynx.

**PGY-2 Pediatric Case: Drowning in a 10-Year-Old**

- **Expected Answers:** ^**16,17**^
  ○ **Sedative:** Ketamine (1–2 mg/kg IV) or etomidate (0.2–0.4 mg/kg IV) for hemodynamic stability.
  ○ **Paralytic:** Succinylcholine (1–2 mg/kg IV) or rocuronium (1 mg/kg IV).
  ○ **Key Considerations:** Anticipate pulmonary edema and ensure ventilator settings account for reduced lung compliance (reducing tidal volume to 6 mL/kg of predicted body weight, optimizing positive end-expiratory pressure (PEEP), and using low fraction of inspired oxygen (FiO_2_).

**PGY-3 Adult Case: Asthma Exacerbation with Impending Respiratory Failure**

- **Expected Answers:** ^**18**^
  ○ **Sedative:** Ketamine (1–2 mg/kg IV), preferred for its broncho-dilatory properties.
  ○ **Paralytic:** Consider rocuronium (1 mg/kg IV) over succinylcholine to avoid worsening hyperkalemia if prolonged hypoxia is present.
  ○ Adjust tidal volumes and respiratory rates post-intubation to avoid air trapping (low tidal volume, slow respiratory rate).

**PGY-3 Pediatric Case: Foreign Body Aspiration in a 6-Year-Old**

- **Expected Answers** ^**19**^ **:**
  ○ **Sedative:** Ketamine (1–2 mg/kg IV) to maintain airway tone and reflexes. Consider awake intubation.
  ○ **Paralytic:** Use cautiously; rocuronium (1 mg/kg IV) only if the decision for intubation is definitive.
  ○ **Adjuncts:** Rigid bronchoscopy backup for foreign body removal, ENT consult, and preparation for surgical airway if obstruction worsens.

#### Diagnostic Craps

**If they roll a total of “2”: STEMI^20^**

- **PGY-1: Inferior STEMI**
  ○ Identification of ST-elevation in leads II, III, and aVF.; Recognition of reciprocal ST-depression in leads I and aVL.
  ○ Understanding the clinical correlation with symptoms such as chest pain and bradycardia due to involvement of the right coronary artery (RCA).
- **PGY-2: Posterior STEMI**
  ○ Recognizing reciprocal changes (ST-depression) in leads V1–V3.
  ○ Using posterior leads (V7–V9) to confirm ST-elevation.
  ○ Understanding the association with RCA or circumflex artery involvement.
  ○ Management adjustments, such as considering posterior infarction in hypotension or bradycardia.
- **PGY-3: Right Ventricular STEMI**
  ○ ST-elevation in V1 (and possibly V4R if right-sided leads are placed).
  ○ Understanding the need to avoid nitrates and diuretics due to preload dependence in RV infarction.
  ○ Management focus on volume resuscitation and cath lab activation.
- ***If “2” is rolled again:***
  ○ What are the initial medications and interventions for this patient? (show EKG per year)
    ▪ Medications and interventions: Aspirin, P2Y12 inhibitor, anticoagulation (heparin), nitroglycerin (avoid for inferior wall myocardial infarction [MI]), and cath lab activation for percutaneous coronary intervention (PCI).
  ○ The patient develops hypotension post-MI. What are possible causes, and how would you manage them?
    ▪ Causes of post-MI hypotension: Free wall rupture, papillary muscle rupture, right ventricle (RV) infarct, cardiogenic shock. Management depends on cause (e.g., fluids for RV infarct, vasopressors, emergent surgery).

**If they roll a total of “3”: Atrial Fibrillation^21^ (AF)**

- **PGY-1 Answer:** Atrial Fibrillation. Irregularly irregular rhythm, no P waves, narrow QRS.
- **PGY-2 Answer:** Rate control (e.g., beta-blockers or calcium channel blockers) vs. rhythm control (antiarrhythmics like amiodarone); decision depends on symptoms and duration of AF (<48 hours for rhythm control).
- **PGY-3 Answer:** Unstable patients require synchronized cardioversion; energy level depends on defibrillator (e.g., 100–200 J biphasic).

**If they roll a total of “4”: Complete (Third-Degree) Heart Block^21^**

- **PGY-1 Answer:** Complete Heart Block. No relationship between P waves and QRS complexes; QRS may be narrow (junctional escape) or wide (ventricular escape).
- **PGY-2 Answer:** Transvenous pacing (TVP); isoproterenol infusion; Atropine typically will not work.
- **PGY-3 Answer:** Ischemia (MI affecting AV node, RCA involved), Lyme late stage, viral myocarditis, degenerative conduction system disease (e.g., Lev’s disease), hyperkalemia, drug toxicity (e.g., beta-blockers, calcium channel blockers, digoxin).

**If they roll a total of “5”: Supraventricular Tachycardia (SVT)^21^**

**EKG Pattern:** Narrow QRS complex, tachycardia with regular rhythm.

- **PGY-1 Answer:** SVT. Vagal maneuvers and Adenosine
- **PGY-2 Answer:** SVT. For refractory SVT, consider synchronized cardioversion (50–100 J).
- **PGY-3 Answer:** Try vagal maneuvers (e.g., ice pack to the face, blowing through a straw). Administer adenosine IV (0.1 mg/kg, max 6 mg; may repeat at 0.2 mg/kg, max 12 mg). If patient is unstable, immediate synchronized cardioversion (0.5–1 J/kg, increase to 2 J/kg if needed).

**If they roll a total of “6”: Hyperkalemia^22^**

- **PGY-1 Answer:** Hyperkalemia. Peaked T waves are the earliest sign; wide QRS and sine wave appear in severe hyperkalemia.
- **PGY-2 Answer:** Stabilize membrane with calcium gluconate, shift potassium intracellularly with insulin + D50, albuterol, and sodium bicarbonate.
- **PGY-3 Answer:** If refractory, initiate dialysis or escalate to veno-venous extracorporeal membrane oxygenation (VV-ECMO) for unstable patients with dysrhythmias.

**If they roll a total of “7”: Ventricular Tachycardia^21^**

- **PGY-1 Answer:** Amiodarone (150 mg IV over 10 mins), procainamide 17 mg/kg, sotalol, lidocaine
- **PGY-2 Answer:** Defibrillation if pulseless; magnesium, isoproterenol or transvenous pacing to increase heart rate and shorten QT interval (target HR ~90–100 bpm), “Overdrive pacing.”
- **PGY-3 Answer:**
  ○ VT: More common in older patients, structural heart disease, or ischemia.
  ○ SVT with aberrancy: More common in younger patients or those with pre-existing bundle branch blocks.
  ○ EKG clues favoring VT:
    ▪ AV dissociation: Look for independent atrial activity (P waves unrelated to QRS).
    ▪ Capture beats: Occasional normal QRS complexes interrupting the wide-complex rhythm.
    ▪ Fusion beats: Hybrid complexes resulting from simultaneous activation of the ventricles by SVT and intrinsic conduction.
    ▪ QRS morphology: Positive or negative concordance across all precordial leads, very wide QRS (>160 ms). Extreme axis deviation (“northwest axis”).

**If they roll a total of “8”: Brugada Syndrome^21^**

- **PGY-1 Answer:** Brugada Syndrome. Coved ST elevation in V1–V3 with RBBB morphology is diagnostic.
- **PGY-2 Answer:** Avoid medications that exacerbate Brugada (e.g., sodium channel blockers). Admit for monitoring and consult electrophysiology.
- **PGY-3 Answer:** Discuss familial screening and genetic testing; some cases require an implantable cardioverter-defibrillator (ICD).

**If they roll a total of “9”: Wolff-Parkinson-White (WPW)^21^**

- **PGY-1 Answer:** WPW. Delta wave with short PR interval and wide QRS is pathognomonic.
- **PGY-2 Answer:** WPW with AF: Avoid AV nodal blockers (e.g., adenosine, beta-blockers); use procainamide or cardioversion.
- **PGY-3 Answer:** Discuss ablation as a definitive treatment; educate patients about activity restrictions until treated.

**If they roll a total of “10”: De Winter’s T-Waves^20^**

- **PGY-1 Answer:** De Winter T-waves.
- **PGY-2 Answer:** De Winter T-waves. Leads V1–V6, as they reflect the anterior/septal wall, which is the region supplied by the left anterior descending artery (LAD).
- **PGY-3 Answer:** Activate the Cath lab immediately because it represents a STEMI-equivalent; start antiplatelet therapy and heparin while ensuring PCI readiness.

**If they roll a total of “11”: Second-Degree AV Block (Type I and II)^21^**

- **PGY-1 Answer:**
  ○ Type I (Wenckebach): Progressive PR prolongation until a QRS is dropped.
  ○ Type II: Fixed PR interval with intermittent dropped QRS complexes.
- **PGY-2 Answer:**
  ○ Make sure they can identify which is type I and type II.
  ○ Indications for pacing: Symptomatic bradycardia, hypotension, or high risk of complete block (e.g., Type II).
- **PGY-3 Answer:**
  ○ Type I and Type II. Reversible causes include ischemia, medications, and metabolic disturbances.

**If they roll a total of “12”:** Identify what is happening with the EKG and give an example of what can cause this:

- **PGY-1 EKG:** S1Q3T3 (not specific), but can be seen with pulmonary embolism (PE)^23^
- **PGY-2 EKG:** Bi-Ventricular Tachycardia: Can be seen with Digoxin Overdose^24^
- **PGY-3 EKG:** TCA Overdose Terminal R wave, widened QRS^25^
  ○ Intraventricular conduction delay: QRS > 100 ms in lead II
  ○ Terminal R wave > 3 mm in aVR or R/S ratio > 0.7 in aVR
  ○ Patients with tricyclic overdose will also usually demonstrate sinus tachycardia secondary to muscarinic (M1) receptor blockade

**Back Up Question: Can use if they repeat a number they already rolled: Atrial Flutter^21^**

- **PGY-1 Answer:** Sawtooth pattern (best seen in II, III, aVF).
- **PGY-2 Answer:** Anticoagulation decision: Use CHA2DS2-VASc score; anticoagulate for score ≥2.
- **PGY-3 Answer:** Perform transesophageal echocardiogram (TEE) to rule out thrombus before cardioversion if onset >48 hours.

#### Play Your Cards Right

##### HEARTS

**ACE:** IV fluids and furosemide (to promote calcium excretion), bisphosphonates, calcitonin^26^

**TWO:** Class Ia (e.g., procainamide) and Class III (e.g., amiodarone) antiarrhythmics since they can worsen sodium channel blockade^25^

**THREE:** Ruptured aneurysm^27^

**FOUR:** Naloxone (Narcan)^28^

**FIVE:** Streptococcus pneumoniae^29^

**SIX:** Intramuscular epinephrine (1:1,000) 0.3 – 0.5 mg (0.3 mL)^30^

**SEVEN:** Needle decompression (with a 14- or 16-gauge needle)^31^

**EIGHT:** Obtain the acetaminophen **(**APAP) level at least 4 hours after ingestion to accurately assess the risk of hepatotoxicity using the Rumack-Matthew nomogram. Levels drawn earlier than 4 hours may be unreliable. NAC should be administered if: the APAP level is above the treatment line on the Rumack-Matthew nomogram for acute overdose, there is delayed presentation (>8 hours) and the APAP level is unknown or concerning for hepatotoxicity, or there are clinical signs of liver injury (elevated transaminases) regardless of APAP level^32^

**NINE:** Benzodiazepines (e.g., lorazepam or diazepam), then Benzos again, then second-line agents (e.g., fosphenytoin, valproic acid, or levetiracetam), and prepare to intubate. If there is no response to second-line therapy, consider continuous infusion anesthetics (e.g., midazolam, propofol, or pentobarbital) and you must secure the airway.^33^

**TEN:** Flumazenil^34^

**JACK:** Any of the following:^35^

- Initial thoracostomy tube drainage > 20 mL/kg, or more than 1500 mL at once
- Output >200 mL/hr for first 3 hours
- Persistent bleeding at rate >7 mL/kg/hour
- Increasing hemothorax on CXR
- Patient hypotensive after adequate blood replacement
- Patient decompensates after initial response to resuscitation

**QUEEN: “**Jefferson bit off a hangman’s thumb”: Jefferson Burst fx (C1), Bilateral facet dislocation, Odontoid (II and III), Atlanto-occipital dissociation, Hangman (C2), flexion Teardrop fracture.^36^

**KING:** Anticoagulation (e.g., heparin or rivaroxaban) and/or thrombolysis if severe^37^

##### DIAMONDS

**ACE:** IV fluids and pain management^38^

**TWO:** Adhesions from previous abdominal surgery^39^

**THREE:** Encephalitis (e.g., herpes simplex virus)^40^

**FOUR:** Insulin or sulfonylurea overdose^41^

**FIVE:** Acute myocardial infarction (MI)^42^

**SIX:** Secure the airway (intubation) and administer antibiotics (e.g., ceftriaxone or cefotaxime)^43^

**SEVEN:** Peptic ulcer disease (PUD)^44^

**EIGHT:** Hydroxocobalamin and sodium thiosulfate, sodium nitrate^45^

**NINE:** Hypertrophic obstructive cardiomyopathy (HOCM)^46^

**TEN:** Nebulized albuterol and/or ipratropium and systemic corticosteroids^47^

**JACK:** IV hydrocortisone and aggressive fluid resuscitation^48^

**QUEEN:** High-flow nasal cannula (HFNC) or CPAP^49^

**KING:** IV dextrose (D25/50 bolus). Rule of 5’s: 36/18 mL^50^

##### CLUBS

**ACE:** Unintentional esophageal intubation^51^

**TWO:** Intussusception^52^

**THREE:** Cytomegalovirus (CMV) infection^53^

**FOUR:** Septic arthritis; Kocher criteria of fever (temperature >101.1°F [>38.4°C]), white blood cell count greater than 12,000/μL (12 × 10^9^/L), Erythrocyte Sedimentation Rate (ESR) greater than 40 mm/hr, and inability to bear weight can be used to assess for septic arthritis^54^

**FIVE:** Prostaglandin E1; Apnea^55^

**SIX:** IV calcium, glucagon, high-dose insulin/glucose, and possibly vasopressors^56^

**SEVEN:** Activated charcoal (if within 1 hour of ingestion) and sodium bicarbonate for alkalinization of urine^57^

**EIGHT:** Simple febrile seizure^58^

**NINE:** Observation can be appropriate for up to 24 hours before intervening if no severe symptoms develop^59^

**TEN:** Doxycycline^60^

**JACK:** Uterine atony^61^

**QUEEN:** Croup (laryngotracheobronchitis)^62^

**KING:** Symptoms include dry mouth, blurred vision, tachycardia, and urinary retention. Treatment is with physostigmine (cholinesterase inhibitor)^63^

##### SPADES

**ACE:** Second intercostal space, midclavicular line, or fifth intercostal space, midaxillary line^64^

**TWO:** Atropine and pralidoxime^65^

**THREE:** Persistent ST elevation in the anterior precordial leads (V1–V6), often with deep Q waves^66^

**FOUR:** Beta-blockers (e.g., propranolol), antithyroid drugs (e.g., methimazole), potassium iodide and supportive care^67^

**FIVE:** Neurogenic shock (due to disruption of sympathetic tone)^68^

**SIX:** Acute liver failure^32^

**SEVEN:** Fomepizole or ethanol^69^

**EIGHT:** Serotonin syndrome, treated with benzodiazepines for sedation and cooling measures, and discontinuation of the offending agent^70^

**NINE:** IV acetazolamide, followed by topical beta-blockers (e.g., timolol) and possible laser therapy (iridotomy). Can add alpha2 agonist as well (Brimonidine)^71^

**TEN:** 4–6 mL/kg of predicted body weight^16^

**JACK:** Deferoxamine^72^

**QUEEN:**100% oxygen via non-rebreather mask or hyperbaric oxygen therapy if severe^73^

**KING:** Air or contrast enema^74^

#### Rapid Response Roulette

**If they land on “1”: VF/VT Arrest in a Known CAD Patient** ^**75**^

1. Immediate defibrillation if the rhythm is shockable.
2. Defibrillation followed by CPR and administration of epinephrine and amiodarone.
3. Consider early coronary reperfusion. (call Code STEMI while resuscitating).
4. Consider ECMO, thrombolytics if you cannot get to a Cath center in an *appropriate time course.*

**If they land on “2”: Asystole Arrest** ^**75**^

1. Continue CPR and provide epinephrine every 3–5 minutes.
2. If no ROSC after 20 minutes, consider discussing with the family for withdrawal of care.
3. An end-tidal carbon dioxide level of 10 mm Hg or less measured 20 minutes after the initiation of advanced cardiac life support accurately predicts death in patients with cardiac arrest associated with electrical activity but no pulse.

**If they land on “3”: Pulseless Electrical Activity (PEA) Arrest** ^**75**^

1. Continue CPR
2. Administer epinephrine every 3–5 minutes
3. Search for a reversible cause (e.g., hypovolemia, tension pneumothorax, PE)
4. Use bedside ultrasound to help find reversible causes (H+Ts)

**If they land on “4”: Hypothermic Cardiac Arrest** ^**76**^

1. Perform prolonged resuscitation efforts (at least 30 minutes)
2. Remove wet clothing
3. Correct hypothermia with active rewarming (warm IV fluids, foley thermometer, bear hugger, heating blankets, chest tube(s), warmed peritoneal/bladder lavage)
4. Consider ECMO if available

**If they land on “5”: TCA Overdose and Cardiac Arrest** ^**25**^

1. Begin CPR immediately and administer epinephrine every 3–5 minutes
2. Secure the airway (BVM, intubation)
3. Give sodium bicarbonate (main antidote)
  IV fluids, vasopressors for hypotension, and consider lipid emulsion therapy (last resort)

**If they land on “6”: Torsades de Pointes** ^**21**^

1. Immediate defibrillation
2. CPR and administer epinephrine every 3–5 minutes
3. Administer 2 g IV push of Magnesium Sulfate

**If they land on “7”: Sepsis in a Diabetic Patient** ^**77**^

1. Immediately start broad-spectrum antibiotics
2. Initiate IV fluids (30 mL/kg bolus)
3. Assess for sources of infection

**If they land on “8”: Neonatal Sepsis** ^**78**^

1. Obtain fingerstick
2. Obtain blood cultures
3. Start antibiotics (e.g., ampicillin & gentamicin)
4. Obtain urinalysis, urine culture, and lumbar puncture

**If they land on “9”: Sepsis in an Immunocompromised Patient** ^**77**^

1. Immediately start broad-spectrum antibiotics
2. Initiate IV fluids (30 mL/kg bolus)
3. Assess for sources of infection
4. Consider antifungal coverage in neutropenic patients

**If they land on “10”: Necrotizing Fasciitis** ^**79**^

1. Start broad-spectrum antibiotics (e.g., piperacillin-tazobactam, clindamycin, vancomycin)
2. Initiate IV fluids (30 mL/kg bolus), initiate vasopressors if necessary (e.g., norepinephrine)
3. Immediate consultation with surgery
4. Obtain CT imaging to assess extent of infection

**If they land on “11”: Blunt Chest Trauma with Hemothorax** ^**80**^

1. Insert a large bore chest tube for drainage
2. Administer blood for hypovolemia
3. Obtain imaging to evaluate the extent of injury

**If they land on “12”: Penetrating Abdominal Trauma** ^**81**^

1. Perform a focused assessment with sonography in trauma (FAST) exam
2. Prepare for surgery if peritonitis or free fluid is noted
3. Initiate resuscitation (e.g., IV fluid, blood products)

**If they land on “13”: Traumatic Brain Injury (TBI)** ^**82**^

1. Airway management with C-spine immobilization
2. Control intracranial pressure (e.g., head of bed elevation, mannitol)
3. Initiate neuroimaging (CT head)

**If they land on “14”: Pelvic Fracture with Shock** ^**83**^

1. Apply pelvic binder over greater trochanters
2. Administer IV fluids and blood products
3. Consult for possible Interventional Radiology (IR) or surgical management

**If they land on “15”: Trauma with Suspected Compartment Syndrome** ^**84**^

1. Measure compartment pressures
2. Release the compartment if elevated (>30) or delta pressure <30
3. Consult orthopedics for fasciotomy if necessary

**If they land on “16”: Burns with Airway Compromise** ^**85**^

1. Secure the airway early (describe technique: fiberoptics ideal)
2. Administer IV fluids using the Parkland formula: 4 x kg x % body surface area (BSA)
3. Provide pain control

**If they land on “17”: Crush Injury with Renal Complications** ^**86**^

1. Administer IV fluids
2. Check Creatine Kinase (CK) level
3. Prepare for possible dialysis
4. Monitor electrolytes closely

**If they land on “18”: Acute Ischemic Stroke** ^**87**^

1. Confirm eligibility for tissue plasminogen activator (tPA). Time window <4.5 hours from symptom onset
2. Initiate IV access and obtain labs
3. Obtain CT/MRI to rule out hemorrhagic stroke

**If they land on “19”: Hemorrhagic Stroke** ^**88**^

1. Control blood pressure (e.g., with Labetalol, Cardine) with systolic BP Goal 140–160
2. Neurosurgical consultation
3. A-line placement
4. Head of bed elevation

**If they land on “20”: Severe Asthma Exacerbation** ^**18**^

1. Administer nebulized albuterol and ipratropium
2. Start systemic steroids
3. Consider magnesium sulfate
4. Consider Noninvasive Positive Pressure Ventilation (NIPPV) & prepare for possible intubation if respiratory failure is imminent

**If they land on “21”: Acute Anaphylaxis** ^**30**^

1. Administer IM epinephrine
2. Ensure airway protection
3. Start IV fluids, antihistamines, and steroids
4. Monitor for progression to shock and consider second dose of epinephrine if symptoms persist

**If they land on “22”: Acute Gastrointestinal Bleed** ^15,89^

1. Start IV fluids and blood products
2. Obtain CBC and coagulation studies
3. Consult gastroenterology for endoscopy
4. If signs of shock persist, consider a Minnesota Tube and/or vasopressors

**If they land on “23”: Suction Assisted Laryngoscopy and Airway Decontamination (SALAD) Intubation** ^**90**^

1. Open mouth placing blade in mouth, place DuCanto catheter into the oropharynx. Begin continuous suction to clear secretions, blood, or vomitus.
2. Continue decontamination of the hypopharynx, followed by insertion of the rigid suction catheter into the proximal esophagus to serve as a continued drain of emesis
3. Reposition the suction catheter to the left corner of the patient’s mouth facilitated by a slight withdrawal and reinsertion of the laryngoscope blade to permit this transit. The laryngoscope blade will now effectively pin the suction catheter in place with this maneuver, as the left portion of the blade contacts the right portion of the suction catheter, securing it in place and holding it out of the path of endotracheal intubation
4. Place bougie/ET tube

**If they land on “24”: Heat Stroke** ^**91**^

1. Immediate cooling with ice packs (body bag), cool IV fluids, and aggressive hydration
2. Cooling blanket, evaporative cooling
3. Avoid shivering (give benzodiazepines), consider sedation if agitation is severe.
4. Monitor for signs of organ failure and prepare for intensive care unit (ICU) admission

**If they land on “25”: Severe Epistaxis** ^**92**^

1. Apply direct pressure for 10–15 minutes
2. Consider decongestant nasal spray (e.g., oxymetazoline) for vasoconstriction
3. If bleeding persists, perform nasal packing, soak in tranexamic acid (TXA), and consult ENT for further management

**If they land on “26”: Alcohol Withdrawal** ^**93**^

1. Start IV benzodiazepines (e.g., lorazepam or diazepam) for sedation and seizure prevention
2. Monitor for signs of delirium tremens
3. Initiate fluids and thiamine supplementation

**If they land on “27”: Thyroid Storm** ^**67**^

1. Hydration with IV fluids and electrolyte correction
2. Beta-Blockers: Propranolol to control tachycardia
3. Antithyroid Meds: Propylthiouracil (PTU) or methimazole
4. Iodine: Lugol’s solution or potassium iodide after thionamides
5. Corticosteroids: Hydrocortisone to inhibit T4 to T3 conversion.
6. Antipyretics: Acetaminophen for fever control
7. Consult Endocrinology and ICU admission

**If they land on “28”: Needle Cricothyrotomy** ^**94**^

1. Clean the area and stabilize the thyroid cartilage
2. Insert a large-bore needle (e.g., 14-gauge) through the cricothyroid membrane at a 45-degree angle caudally
3. Aspirate air to confirm tracheal entry
4. Attach a catheter to a jet ventilation system or bag-valve mask adaptor

**If they land on “29”: DKA (Diabetic Ketoacidosis)** ^**95**^

1. IV fluid bolus over the first hour to restore intravascular volume (20 ml/kg)
2. Initiate regular insulin drop at 0.1 units/kg/hour after starting fluids (continue drip until the anion gap closes, not just when glucose normalizes)
3. Check potassium levels prior to insulin initiation: If potassium < 3.3 mEq/L: Hold insulin and replace potassium. If potassium 3.3–5.0 mEq/L: Add potassium (20–40 mEq/L) to IV fluids. If potassium > 5.0 mEq/L: Monitor closely and do not supplement
4. Monitor for cerebral edema: watch for signs (e.g., headache, bradycardia, altered mental status). Treat with mannitol or hypertonic saline if suspected

**If they land on “30”: Wayne Pneumothorax catheter** ^**96,97**^

1. Acceptable sites: 2^nd^ intercostal space, midclavicular line (anterior approach) or the 4^th^–5^th^ intercostal space mid-to-anterior axillary line (lateral approach)
2. Confirm the pneumothorax location with chest X-ray or ultrasound, if available
3. Infiltrate lidocaine at the insertion site, including down to the pleura
4. Attach a syringe filled with a small amount of sterile saline to the provided needle. Insert the needle perpendicular to the chest wall (or at a slight angle if using the lateral approach). Advance slowly while aspirating with the syringe until air bubbles are seen in the saline (indicating entry into the pleural space)
5. Stabilize the needle and remove the syringe. Insert the guidewire through the needle into the pleural space, ensuring it passes smoothly without resistance. Remove the needle, leaving the guidewire in place
6. Use the provided dilator to enlarge the soft tissue tract over the guidewire. Advance the dilator only to the level of the pleural space and remove it immediately after dilation
7. Thread the Wayne catheter over the guidewire and advance it into the pleural space. Ensure the side holes of the catheter are fully within the pleural space
8. Remove the guidewire and confirm airflow or fluid drainage from the catheter. Secure the catheter with sutures or a securement device. Connect the catheter to a Heimlich valve, water seal system, or suction device, as clinically indicated
9. Obtain a chest X-ray to ensure proper catheter placement and pneumothorax resolution

**If they land on “31”: Lateral Canthotomy** ^**98**^

1. Make sure no globe rupture
2. Name the Indications**:** Perform in cases of orbital compartment syndrome with signs of vision-threatening elevated intraocular pressure (IOP), such as decreased vision, proptosis, or afferent pupillary defect
3. Lidocaine with epinephrine for local anesthesia. Use clamps to stop bleeding/hold pressure.
4. Using scissors, make a horizontal incision at the lateral canthus, cutting through the skin and lateral canthal tendon. Extend the incision approximately 1–2 cm, separating the eyelids
5. Identify the inferior crus of the lateral canthal tendon (running toward the orbital rim).Use scissors to cut the inferior crus, freeing tension on the globe and reducing IOP
6. If necessary, release the superior crus for further decompression
7. Reassess intraocular pressure and clinical improvement (e.g., reduced proptosis, improved visual acuity)

**If they land on “32”: T-piece** ^**99**^

1. Ensure it is connected to a gas source (blended oxygen/air, starting at 21% FiO_2_ for term neonates or 30% for preterm)
2. Set initial pressures: Peak Inspiratory Pressure (PIP): 20–25 cm H_2_O
3. Positive End-Expiratory Pressure (PEEP): 5 cm H_2_O
4. Confirm gas flow: 10L/min and Rate: 40–60 breaths per minute

**If they land on “33”: Nursemaids Elbow Reduction** ^**100**^

1. Supination-Flexion Method:
  1. Hold the child’s elbow and wrist.
  2. Supinate (rotate palm upward) the forearm while simultaneously flexing the elbow completely (most common)
2. Hyperpronation Method:
  1. Hold the child’s elbow at a 90-degree angle
  2. Firmly grasp the child’s wrist and pronate (rotate) the forearm downward while maintaining pressure on the radial head

**If they land on “34”: Myxedema Coma** ^**101**^

1. Intubate if AMS/secure airway
2. Start IV fluids (isotonic or with dextrose if hypoglycemic).
3. Hormonal Replacement: IV levothyroxine: 300–500 mcg bolus, then 50–100 mcg daily
4. Treat Hypotension: Use IV fluids; add vasopressors if unresponsive to fluids
5. Address adrenal insufficiency: IV hydrocortisone 100 mg every 8 hours until adrenal crisis is ruled out
6. Antipyretics: Acetaminophen for fever control
7. Rewarm: use passive warming with blankets – avoid aggressive rewarming to prevent vasodilation
8. Identify triggers: Treat infections or other precipitation factors (e.g., sepsis, MI)

**If they land on “35”: Anterior Shoulder Dislocation Reduction** ^**102**^

1. Give Intra-articular lidocaine injection (10–20 mL)
2. Kocher Method
  1. Adduct the arm against the body
  2. Flex the elbow to 90 degrees
  3. Slowly externally rotate the arm until resistance is felt, then lift the elbow anteriorly while internally rotating
3. Scapular manipulation: Rotate the scapula medially by pressing the inferior tip while pushing the superior portion laterally
4. Apply traction/countertraction
5. Milch Technique
  1. Patient supine, gently abduct the arm to 90 degrees and externally rotate
  2. Apply gentle traction and pressure over the humeral head with the opposite hand

**If they land on “36”: SBP (Spontaneous Bacterial Peritonitis) and Paracentesis** ^**103**^

1. Stabilize the patient (address hypotension with IV fluids such as albumin or crystalloid)
2. Empirically start IV antibiotics (e.g., cefotaxime 2 g IV every 8 hours or an equivalent third-generation cephalosporin)
3. Place the patient supine with the head of the bed slightly elevated (20–30°) Identify the puncture site: typically 2 cm below the umbilicus in the midline (avoiding large vessels) or in the left or right lower quadrant, lateral to the rectus sheath
4. Insert the needle perpendicular to the skin, advancing slowly while aspirating. Stop when ascitic fluid is aspirated
5. Collect fluid in sterile tubes for analysis (cell count, culture, albumin, and total protein). Monitor for complications (e.g., bleeding, infection, or bowel perforation). Ascitic fluid polymorphonuclear leukocytes (PMNs) ≥ 250 cells/mm_3 confirms the diagnosis. Send fluid for culture to identify the pathogen (commonly E. coli or Klebsiella)

**If they land on “0”: Pediatric Lumbar Puncture** ^**104**^

1. Obtain Consent: Explain the procedure, risks, and benefits to the parents
2. Gather Supplies: PPE, iodine, Spinal needle (22–25 gauge with stylet, usually 1.5 inches for neonates). Sterile collection tubes (labeled 1–4)
3. Place the infant on their side, with the head and knees gently flexed toward the chest to open the intervertebral spaces. Flexed Lateral Decubitus Position (preferred)
4. Palpate the iliac crests to locate the L4 vertebral level. Insert the needle at the L3–L4 or L4–L5 interspace
5. Patient should be on pulse ox/monitoring - monitor for apnea

**If they land on “00”: Cardiac Tamponade** ^**105**^

1. Place patient in supine position with head elevated to 30–45 degrees (semi-recumbent) to allow fluid to collect in the most dependent area of the pericardium (near the apex). Identify the subxiphoid area (just below the xiphoid process)
2. Insert the needle at a 30–45° angle to the abdominal wall, aiming toward the left shoulder. Use continuous aspiration with the syringe to detect fluid. Advance slowly and stop once fluid is aspirated. Aspirate as much fluid as possible to relieve tamponade. If connected to a 3-way stopcock, maintain sterility while removing fluid
3. Confirmed Success: Improvement in blood pressure, heart sounds, and symptoms. Ultrasound confirmation (decrease in effusion size). Alternate techniques: Apical Approach: Insert the needle at the fifth or sixth intercostal space, left midclavicular line. Use ultrasound for guidance to avoid injury to the lung or coronary arteries
4. Parasternal Approach: Insert at the left parasternal region in the fourth or fifth intercostal space, aiming toward the right shoulder. If the needle touches the myocardium: A current of injury pattern (ST elevation) may be seen on ECG if connected to the needle

**Extra Scenarios**

**Septic Joint/Arthrocentesis** 106

1. Preparation: Assemble sterile equipment including a needle (18–21 gauge), syringe, antiseptic, and sterile drapes
2. Positioning: Position the patient with the knee slightly flexed to relax the joint capsule
3. Sterile technique: Clean the skin over the knee (usually the medial aspect of the knee joint) with antiseptic solution and drape the area
4. Anesthesia: Administer local anesthesia with lidocaine around the site of aspiration
5. Needle insertion: Insert the needle in the inferior-medial aspect of the patella, aiming toward the femur, and aspirate to remove fluid. Consider using ultrasound
6. Fluid Collection: Once the effusion is aspirated, remove the needle and apply a sterile dressing to the site. Send the fluid for analysis (e.g., cell count, Gram stain, culture)

**Subclavian Central Line** 107

1. Using the needle, insert at a 30-degree angle just below the clavicle and advance toward the midline, aiming for the sternal notch
2. Once venous blood return is noted, remove the needle and introduce a guidewire through the needle

**Massive Transfusion Protocol (MTP)** ^108^

1. Assign someone to get blood
2. Balanced ratio of packed red blood cells, fresh frozen plasma, and platelets (commonly 1:1:1)
3. Calcium replacement to counteract citrate-induced hypocalcemia
4. Use of thromboelastogram (TEG) or rotational thromboelastogram (ROTEM) for coagulation guidance
5. Maintain normothermia with warming blankets and fluid warmers

#### Diagnostic Craps

**Inferior STEMI**

**Posterior STEMI**

**Right Ventricular STEMI**

**Atrial Fibrillation**

**Complete Heart Block**

**Supraventricular Tachycardia**

**Hyperkalemia**

**Ventricular Tachycardia (monomorphic)**

**Ventricular Tachycardia (polymorphic)**

**Brugada Syndrome**

**Wolff-Parkinson-White**

**De Winter’s T-Waves**

**Second Degree Block Type 1**

**Second Degree Block Type 2**

**S1Q3T3**

**Bi-Ventricular Tachycardia (Digoxin)**

**TCA Overdose**

**Atrial Flutter**
